# Efficient community detection in multilayer networks using boolean compositions

**DOI:** 10.3389/fdata.2023.1144793

**Published:** 2023-08-23

**Authors:** Abhishek Santra, Fariba Afrin Irany, Kamesh Madduri, Sharma Chakravarthy, Sanjukta Bhowmick

**Affiliations:** ^1^Department of Computer Science and Engineering, University of Texas at Arlington, Arlington, TX, United States; ^2^Department of Computer Science and Engineering, University of North Texas, Denton, TX, United States; ^3^Department of Computer Science and Engineering, The Pennsylvania State University, State College, PA, United States

**Keywords:** multilayer network, community detection, network decoupling, homogeneous networks, boolean combination

## Abstract

Networks (or graphs) are used to model the dyadic relations between entities in complex systems. Analyzing the properties of the networks reveal important characteristics of the underlying system. However, in many disciplines, including social sciences, bioinformatics, and technological systems, multiple relations exist between entities. In such cases, a simple graph is not sufficient to model these multiple relations, and a multilayer network is a more appropriate model. In this paper, we explore community detection in multilayer networks. Specifically, we propose a novel *network decoupling strategy* for efficiently combining the communities in the different layers using the Boolean primitives AND, OR, and NOT. Our proposed method, network decoupling, is based on analyzing the communities in each network layer individually and then aggregating the analysis results. We (i) describe our network decoupling algorithms for finding communities, (ii) present how network decoupling can be used to express different types of communities in multilayer networks, and (iii) demonstrate the effectiveness of using network decoupling for detecting communities in real-world and synthetic data sets. Compared to other algorithms for detecting communities in multilayer networks, our proposed network decoupling method requires significantly lower computation time while producing results of high accuracy. Based on these results, we anticipate that our proposed network decoupling technique will enable a more detailed analysis of multilayer networks in an efficient manner.

## 1. Introduction

The relations among entities in social and technological systems can be represented as networks (or graphs), where each relation is an edge and each entity is a vertex. However, in reality, most systems are defined, not by a single, but by multiple types of relations. For example, a group of friends may be connected through multiple social networking platforms such as Facebook, Twitter as well as by email. Technological networks such as transportation networks can consist of airline, train, or bus routes. To accommodate multiple types of relations, the systems are modeled as a set of networks. This set of networks, each representing a different relation, forms a multilayer network. Here, we focus on *homogeneous multilayer networks* (also known as *multiplexes*), where each network is composed of the same set of nodes, but the structure of the network changes, based on the respective relations.

The typical approach to analyzing multilayer networks is to combine all, or a subset, of the layers to form a single network (here termed as a *composed network*), and then apply the existing analysis algorithms for single networks. Given an undirected and unweighted network, the layers can be combined using the primitive Boolean operations AND, OR and NOT.

### 1.1. Motivation

Most current multilayer analysis approaches implicitly assume that the multilayer network is mapped to only one composed network. In real life scenarios, however, queries on multilayer networks can require testing over multiple composed networks formed from different combinations of the layers. As an example, consider a company deciding which routes (airplane, train, road, etc.) to use for transporting material, under limited resources. They may want to select combination of which two routes would give the most connectivity. If the relations with respect to each condition is represented as a layer, then (n2)≈n2 composed networks have to be analyzed, where *n* is the number of layers. Another example would be a city trying to do a risk-benefit analysis of which businesses and public places to re-open after lockdown. In this case, they would want to identify the subset of places that maximizes the benefits but lowers the risks. Assuming that each layer represents the possible social interactions at each place, all possible combinations of the layers have to be analyzed. Here the number of tests would be 2^*n*^.

These examples demonstrate that the number of composed networks increases exponentially with the layers of the multiplex, and analyzing the entire set is computationally expensive. To date, as discussed in Section 2.2, most multilayer analysis is based on creating *only one* composed network, formed by combining all or a subset of the layers using an OR operation. *The problem of efficiently creating different composed networks through varying combinations of layers and operations has been rarely addressed*.

### 1.2. Our contribution

Analysis of the different composed networks will incur some redundant computations, since different composed networks can have some of the layers in common. *We propose to reduce the number of redundant computations through a novel method of network decoupling*. In network decoupling, each individual network layer is analyzed independently once and only once, and then the results are combined as required. Network decoupling (analyze and then combine) has several advantages as follows;

- *Computational efficiency*. Network decoupling reduces the time for analysis. Analyzing each layer separately helps in identifying the relevant edges per layer, and reduces the number of edges to combine.
- *Flexibility of combination*. Network decoupling facilitates incorporating changes to the multilayer network, such as addition of a new layer or change in the structure of an old layer. Using decoupling, only the added or changed layer has to be analyzed, and combined as required.
- *Reduced information loss*. When analyzing a composed network, it is challenging to understand how the individual layers contributed to the analysis. Using network decoupling, since each layer is analyzed individually, their respective contributions can also be identified.

### 1.3. Identifying communities in multilayer networks

**Figure 1:**
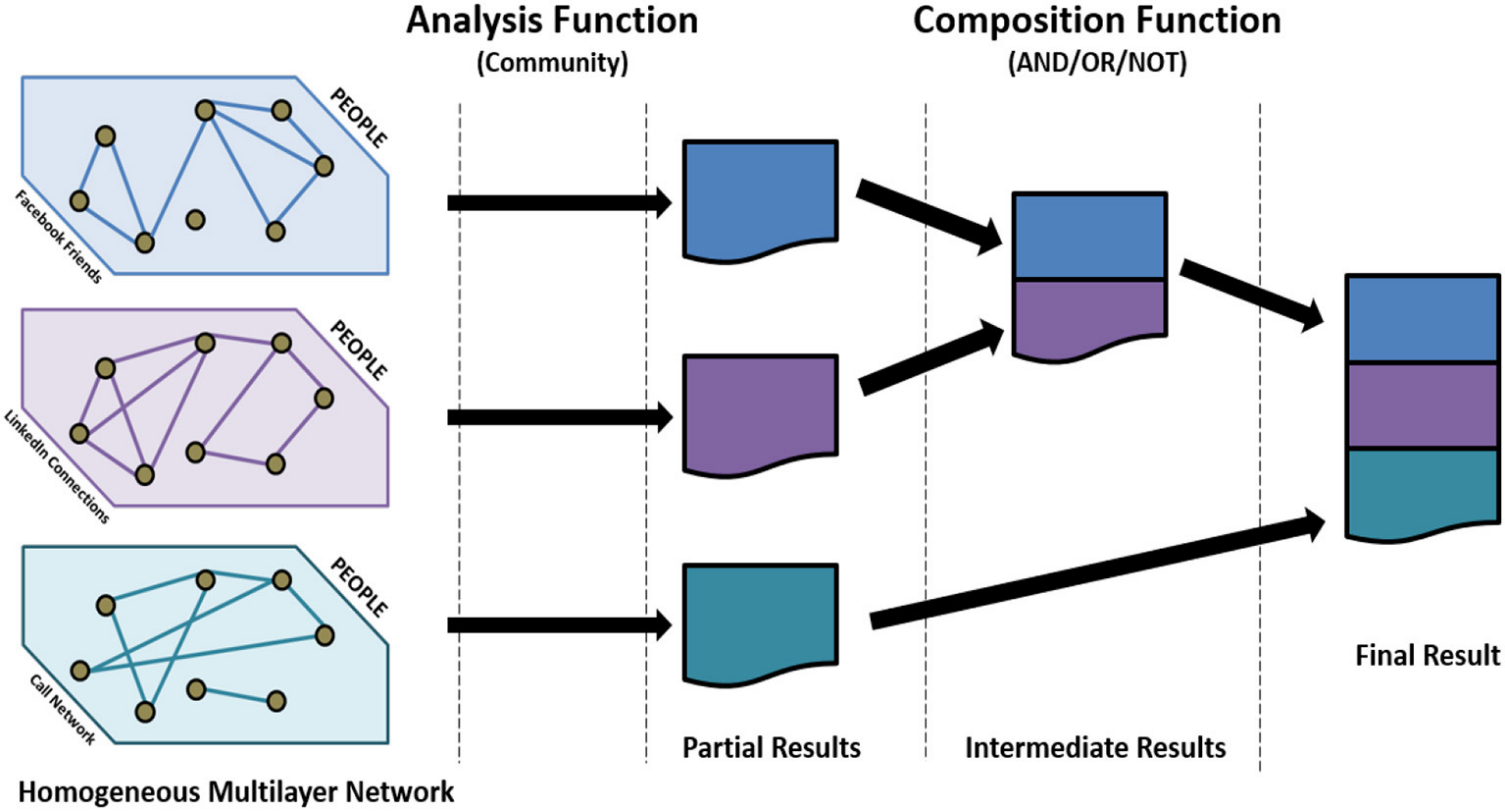
Network decoupling. Each layer is analyzed separately and then the results are aggregated.

Here, we focus on computing communities in multilayer networks using decoupling. Communities are groups of tightly connected nodes. Using network decoupling, we first find the communities in each individual network and then develop aggregation functions, such that the communities from each layer can be combined to produce the communities in the composed network (Figure 1). *The primary challenge is to design appropriate aggregation functions, such that the communities obtained using network decoupling are similar to those obtained by applying community detection on the composed network*^1^. Formally, our problem can be stated as follows;

### 1.4. Problem statement

Given a set of layers *G*_1_, *G*_2_, …, *G*_*x*_, that are combined using a Boolean operation ⊕ to form the composed network, and a community detection algorithm *COMM*, that is used to find communities, develop an aggregation algorithm Π, such that


$$COMM\left(\overset{x}{\underset{i=1}{⊕}}\left(G_{i}\right)\right)\approx \Pi_{i=1}^{x}\left(COMM\left(G_{i}\right)\right)$$

In other words, we aim to find an aggregation algorithm Π, such that *the results of finding the communities in the individual layers and then aggregating them via* Π*, should be similar to the communities obtained from the composed network where the layers are combined using the Boolean operator* ⊕. Developing the aggregation algorithm is challenging, since the structure of the composed network can change after combination, and the aggregation process has to account for that change when combining the communities.

## 2. Overview of communities in multilayer networks

We provide an overview of multilayer network, creation, type of communities in MLN and how boolean operations can be used to combine the layers.

### 2.1. Creating multilayer networks

A multi-relational data set, can be represented as a homogeneous multilayer network. Each feature is represented as a separate layer. The set of entities remain the same in each layer. The edges, based on relations between the entities, change across the layers with respect to the corresponding feature.

We use the Internet Movie Database (IMDb) to illustrate how a multiplex is constructed. The IMDb is an online database that contains information on television programs and movies including actors, directors, genre, and year of release (IMDB-2018, 2018). We create a multiplex where the entities represent actors and two actors are connected to each other if they have acted in the same movie. Each layer in the multiplex represents a movie genre, such as comedy, drama, action, etc.

**Figure 2:**
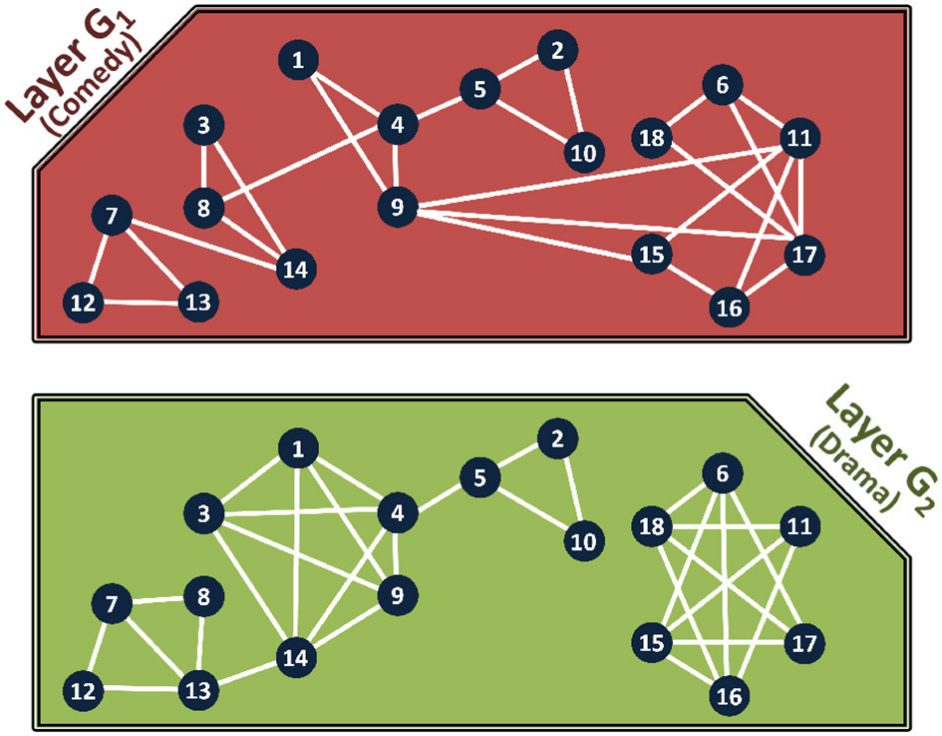
IMDb multiplex for co-actors with 18 actors and two genres: comedy and drama.

An example of the multilayer network is given in Figure 2. We show two genres, comedy (*f*^1^) and drama (*f*^2^) to form the two layers, *G*_1_ and *G*_2_, respectively, and modeled the co-actor relationship among 18 actors (denoted by nodes numbered from 1 to 18). Two actors are connected if they acted in movies of the same genre. Note that although the same 18 actors are present in both layers, the structure varies due to the difference in relations. By taking the information from the two networks together we can gain interesting insights to the data. For example, actors *I*_3_ and *I*_8_ have never worked together in a drama, but have worked together in a comedy. Also, observe that the actors *I*_4_ and *I*_14_ have the most connections in the drama genre, whereas actors *I*_9_, *I*_10_, and *I*_11_ are the nodes with the most connections in comedy.

### 2.2. Pillar and semi-pillar communities in multilayer networks

**Figure 3:**
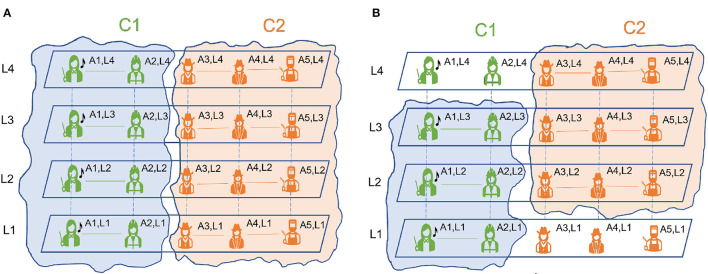
Pillar **(A)** and semi-pillar community structure **(B)**.

In recent literature, Hanteer (2020) and Braun et al. (2021) have differentiated between the id of the entities and how they appear in each layer. Specifically, in Magnani et al. (2021) *an actor* is defined as an entity that can represent a particular person, animal, organization, city, country etc. In Figures 3A, B, there are five actors A1, A2, A3, A4 and A5 representing five different individuals.

We also differentiate between the actor and their existence in a layer. *A node* is the existence of a given actor in a given layer. The same actor present in different layers represents different nodes. In Figures 3A, B, for each of the five actors A1, A2, A3, A4 and A5, there is one node present in each layer. For example: for actor A1 we have nodes A1L1, A1L2, A1L3, A1L4 present in layers L1, L2, L3 and L4, respectively. The node in layer *k* representing actor *i*, is denoted as $u_{k}^{i}$. Using these definitions communities in multilayer networks are defined in Magnani et al. (2021) as follows;

#### 2.2.1. Pillar community

The pillar community extends across all the layers. For each actor that belongs to a community, all the nodes belonging to the actor must belong to the same community. An example of pillar community is presented in Figure 3A.

#### 2.2.2. Semi-pillar community

The semi-pillar community extends across k layers where 2 ≤ *k*<*N*_*L*_. Here, *N*_*L*_ is the total number of layers. As before, for each actor that belongs to a community, all the nodes belonging to the actor must belong to the same community for k layers. We see a structure of semi-pillar community in Figure 3B.

### 2.3. Community detection in composed networks

**Figure 4:**
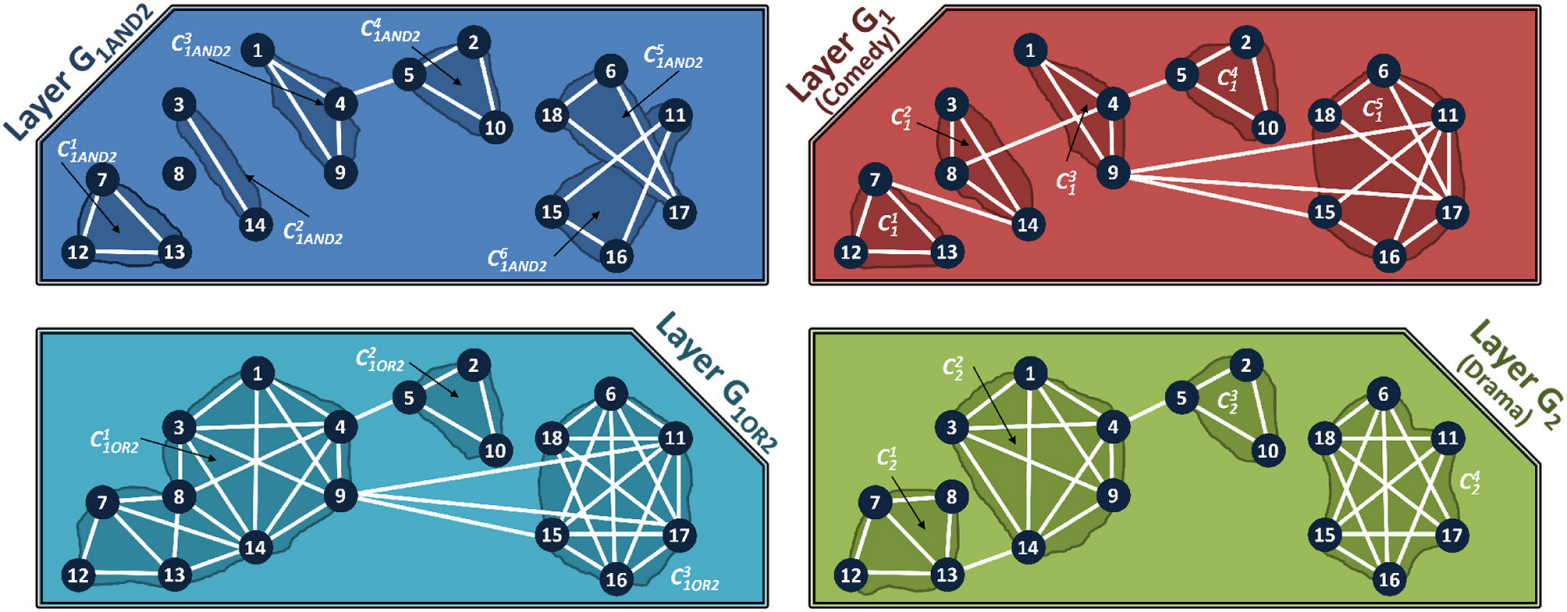
Composed Layer Communities of the Multiplex shown in Figure 2. **Top Left**: Communities in AND composed layer. **Bottom Left**: Communities in OR composed Layer. **Top Right**: Communities in Comedy layer of the IMDb Multiplex. **Bottom Right**: Communities in Drama layer of the IMDb Multiplex.

We now demonstrate how the different layers of the multiplex can be combined, and how communities are detected in them. Figure 4 shows the communities for the composed layers, *G*_1*AND*2_ (AND composition) and *G*_1*OR*2_ (OR composition) for multiplex in Figure 2.

*Communities in AND-Composed Layers*. AND composition allows users to find communities that are related across all the features. Algorithm 1 (termed *C-SG-AND*) shows the steps of finding communities in the composed network, using a standard community detection algorithm such as Infomap (Bohlin et al., 2014). In an AND composition, the composed network is formed of the edges that are common to all the networks, and then the communities are found in the composed network. Some questions that can be addressed by the AND composition are (based on data sets in Section 4).

- Groups of actors who have expertise in working together in *both* comedies and dramas (IMDb multiplex).
- Authors who have co-authored a paper published in *all of these* conferences: ICDM, SIGMOD and VLDB (DBLP multiplex).
- Accidents that have similar conditions for *all of these* features: light conditions, weather conditions, road conditions, and speed limit (Accident Multiplex).

**Algorithm 1:** Algorithm for C-SG-AND.

**Require:** Layers *G*_1_, *G*_2_, …*G*_*x*_
**Ensure:** return $L_{1,2,\ldots ,x}^{AND}$ - a list of communities
1: *G*_1*AND*2…*ANDx*_ ← {*G*_1_ AND *G*_2_ …AND *G*_*x*_} { *G*_1*AND*2…*ANDx*_ contains edges that exist in all the networks *G*_1_, *G*_2_, …, *G*_*j*_.}
2: $L_{1,2,\ldots ,x}^{AND}$ = COMM(*G*_1*AND*2…*ANDx*_) {Find communities in *G*_1*AND*2…*ANDx*_.}

*Communities in OR-composed layers*. OR-composition forms a composed network that includes an edge if it appears in at least one of the layers. Algorithm 2 shows the steps of this single network based community detection using the OR operation, termed as *C-SG-OR*. In an OR composition, the composed network is formed of the union of the edges from all the networks, and then the communities are found in the composed network. Examples of queries that can be addressed by the OR composition are;

- Actors who have acted together in either a comedy *or* drama (IMDb multiplex).
- Authors who have co-authored a paper published in *at least one* conference (DBLP multiplex).
- Accidents that have *at least one* condition in common (Accident Multiplex).

**Algorithm 2:** Algorithm for C-SG-OR.

**Require:** Layers *G*_1_, *G*_2_, …*G*_*x*_
**Ensure:** return $L_{1,2,\ldots ,x}^{OR}$ - a list of communities
1: *G*_1*OR*2…*ORx*_ ← {*G*_1_ OR *G*_2_ …OR *G*_*x*_} { *G*_1*OR*2…*ORx*_ contains edges that are in at least one of the networks *G*_1_, *G*_2_, …, *G*_*x*_.}
2: $L_{1,2,\ldots ,x}^{OR}$ = COMM(*G*_1*OR*2…*ORx*_) {Find communities in *G*_1*OR*2…*ORx*_.}

**Algorithm 3:** Algorithm for CV-AND.

**Require:** Communities from layers *G*_*i*_ and *G*_*j*_: COMM(*G*_*i*_) = {$C_{i}^{1}\left(V_{i}^{1},E_{i}^{1}\right)$, $C_{i}^{2}\left(V_{i}^{2},E_{i}^{2}\right)$, …, $C_{i}^{x}\left(V_{i}^{x},E_{i}^{x}\right)$}, COMM(*G*_*j*_) = {$C_{j}^{1}\left(V_{j}^{1},E_{j}^{1}\right)$, $C_{j}^{2}\left(V_{j}^{2},E_{j}^{2}\right)$,..., $C_{j}^{y}\left(V_{j}^{y},E_{j}^{y}\right)$}
**Ensure:** return $L_{i,j}^{CV-AND}$ - a list of communities
1: $L_{i,j}^{CV-AND}=\emptyset$ {Initialize the set of communities to empty set.}
2: **for** each community pair say, $C_{i}^{p}$ and $C_{j}^{q}$ **do**
3: $C_{i,j}^{p,q}$ =($V_{i}^{p}$ ∩ $V_{j}^{q}$) {Create new combined community by taking the common vertices of every pair of communities.}
4: $L_{i,j}^{CV-AND}$ = $L_{i,j}^{CV-AND}$ ∪ $C_{i,j}^{p,q}$ {Add new community to the set of communities.}
5: **end for**

#### 2.3.1. Bridge edges

We term the external edges that connect two communities as *bridge edges*. Formally, if there exists an edge, $\left(u_{k}^{i},u_{k}^{j}\right)$ such that $u_{k}^{i}$ ∈ $C_{k}^{m}$ (community *m* in layer *k*) and $u_{k}^{j}$ ∈ $C_{k}^{n}$, (community *n* in layer *k*) where *m*≠*n*, then this edge is a bridge edge. In the AND and OR composed-layer of Figure 4, the actors *I*_1_ and *I*_5_ belong to different communities, but are connected by a bridge edge. The actors *I*_9_ and *I*_15_ have a bridge edge in the OR composed network, but not in the AND-composed network.

The AND composed networks have smaller, but more communities, than the OR composed networks. In general, the communities in the AND composed layer form subsets of the communities in the OR composed layer. We will leverage this property to develop our aggregation algorithms.

## 3. Materials and methods

The traditional method of first forming the composed network and then analyzing can be computationally expensive when multiple composed networks have to be considered. This approach can lead to redundant operations. Consider a multiplex with four layers *G*_1_, *G*_2_, *G*_3_, and *G*_4_. Also consider two composed networks formed from the multiplex, namely, *G*_1*AND*2*AND*3_ and *G*_1*AND*2*AND*4_. In this case, the composed layer related to *G*_1*AND*2_ remains unchanged, but has to be recomputed, leading to extra computations.

We propose network decoupling for efficient community detection on multiplex networks. The communities in each layer are identified separately and the results are then aggregated to obtain the results with respect to the composed network. Figure 4 shows the communities in each layer of the IMDb network.

The challenge is to develop aggregation algorithms, that can correctly aggregate the communities from each of the layers to obtain the communities over the composed network. We now present the aggregation methods for AND and OR composition. For ease of understanding we will discuss the algorithms with respect to two layers. Note, however, that our algorithms can be easily extended to multiple layers.

### 3.1. Community detection in composed networks formed using the AND operation

We presented a vertex-based method for finding communities in composed networks formed using AND operations (termed as *AND_composed network*) in Santra et al. (2017). We first discuss this work for completeness. Our algorithm relies on finding self preserving communities.

#### 3.1.1. Self preserving communities

A community is self preserving if the vertices in it are *so tightly connected* such that any connected subset of the vertices will form a smaller community rather than joining an existing larger community. Formally, consider a graph *G*, containing a community whose vertices are given by the set *C*. Consider a subset of vertices *C*_*s*_⊂*C*, such that the subgraph induced by *C*_*s*_ is connected, and |*C*_*s*_|≥3. Remove the subgraph induced by the set of vertices *C*\*C*_*s*_ from the original graph, and then compute the communities again on the new graph Ḡ. If for *all subsets* *C*_*s*_⊂*C*, *C*_*s*_ forms separate community(ies) rather than being fully or partially merged with other communities, then *C* is a self-preserving community. Algorithm 4 outlines the steps to detect all self-preserving communities of a given graph.

**Algorithm 4:** Algorithm for detecting self-preserving communities.

**Require:** Graph, *G*(*V, E*)
**Ensure:** return *SPC*- a set of self-preserving communities
1: *SPC* = ∅ {Initialize the set of self-preserving communities to an empty set.}
2: *C* = *COMM*(*G*)
3: **for** each community *C*_*p*_(*V*_*p*_, *E*_*p*_) ∈ *C* **do**
4: *sp*_*check* = *true*
5: **for** each *V*_*s*_ ⊂ *V*_*p*_, where |*V*_*s*_| ≥ 3 **do**
6: *E*_*s*_ = ∅
7: **for** each edge (*u, v*) ∈ *E*_*p*_ **do**
8: **if** *u* ∈ *V*_*s*_ and *v* ∈ *V*_*s*_ **then**
9: *E*_*s*_ = *E*_*s*_ ∪ {(*u, v*)}
10: **end if**
11: **end for**
12: **if** *G*(*V*_*s*_, *E*_*s*_) is a connected graph **then**
13: *V*′ = *V* \ (*V*_*p*_ \ *V*_*s*_)
14: *E*′ = ∅
15: **for** each edge (*u, v*) ∈ *E* **do**
16: **if** *u* ∈ *V*′ and *v* ∈ *V*′ **then**
17: *E*′ = *E*′ ∪ {(*u, v*)}
18: **end if**
19: **end for**
20: *C*′ = *COMM*(*G*′(*V*′, *E*′)) {for each connected subset of at least 3 vertices from a given community, remove the subgraph induced by the vertices not present in the chosen subset from the original graph, and then find the communities.}
21: **for** each *C*_*r*_(*V*_*r*_, *E*_*r*_) ∈ *C*′ **do**
22: **if** (*V*_*r*_∩*V*_*p*_ ≠ ∅ and *E*_*r*_∩*E*_*p*_ ≠ ∅) and (*V*_*r*_\*V*_*p*_ ≠ ∅ and *E*_*r*_\*E*_*p*_ ≠ ∅) **then**
23: *sp*_*check* = *false*
24: break
25: **end if**
26: **end for** {check if there exists a new community that has partial overlap with the given community (*C*_*p*_). If yes, then this will mean that there exists a subgraph of the given community denoted by *G*(*V*_*s*_, *E*_*s*_) that merges fully or partially with other communities, and hence *C*_*p*_ is not self-preserving.}
27: **end if**
28: **end for**
29: **if** *sp*_*check* = *true* **then**
30: *SPC* = *SPC* ∪ {*C*_*p*_}
31: **end if**
32: **end for**

A self-preserving community indicates that the community is loosely connected with the remainder of the network. Removing parts of the community will not change the structure or composition of the *other communities* in the graph. Thus, the subgraph forming a self-preserving community is not affected by changes to the remainder of the network. The communities in Layer 2 in Figure 4, are all self-preserving, as tested by Louvain (Blondel et al., 2008) and Infomap (Bohlin et al., 2014). That is, every community is tightly connected and for none of the communities, there does not exist any subset of vertices, which when removed from the original graph cause the original community to lose its tightness and merge fully or partially with other communities ^2^.

When two layers are combined using an AND operation, then certain edges are deleted from each layer, and thus from certain communities. If the communities are self preserving, then this deletion will only affect that community and not the others. This is the main result of Santra et al. (2017), which is *if the communities from the layers are self preserving, then the communities of the AND-composed graph can be obtained by taking the intersection of the vertices of the communities from the individual layers*.

#### 3.1.2. Vertex based intersection (CV-AND)

In our paper (Santra et al., 2017), we proposed intersecting the communities based on their vertices. We term this algorithm as *CV-AND* (see Algorithm 3). Here we consider pairs of communities, one from the first layer and the other from the second layer, and obtain the new community by taking the common vertices among the pair. The primary drawback of this method is that if the communities are not self preserving, the results may not be accurate.

As an example, consider the community $C_{1}^{5}$ in the comedy layer of the network (Figure 4), which is made of the nodes {*I*_6_, *I*_11_, *I*_15_, *I*_16_, *I*_17_, *I*_18_}. This community is not self preserving, as when the subgraph induced by the subset of nodes {*I*_6_, *I*_16_} is removed the layer *G*_1_, then its intra-community edge connectivity becomes less tight as compared to its connectivity with other communities and $C_{1}^{5}$ gets merged with $C_{1}^{3}$ to form one single community, thus violating the condition of self-preservation. In this case, applying CV-AND on community $C_{1}^{5}$ from the comedy layer and community $C_{2}^{4}$ from the drama layer, which have the same vertices, gives one large community, { *I*_6_, *I*_11_, *I*_15_,*I*_16_,*I*_17_,*I*_18_}. In reality, as seen in Figure 4, two separate communities are formed, { *I*_6_,*I*_17_,*I*_18_} and { *I*_11_, *I*_15_,*I*_16_}. This is a *subtle but important difference* because the community id determines whether two entities are similar. If two disconnected groups of vertices are placed in the same community (as is possible when using CV-AND), then, two dissimilar groups are marked to be similar, which is incorrect.

#### 3.1.3. Edge based intersection (CE-AND)

**Figure 5:**
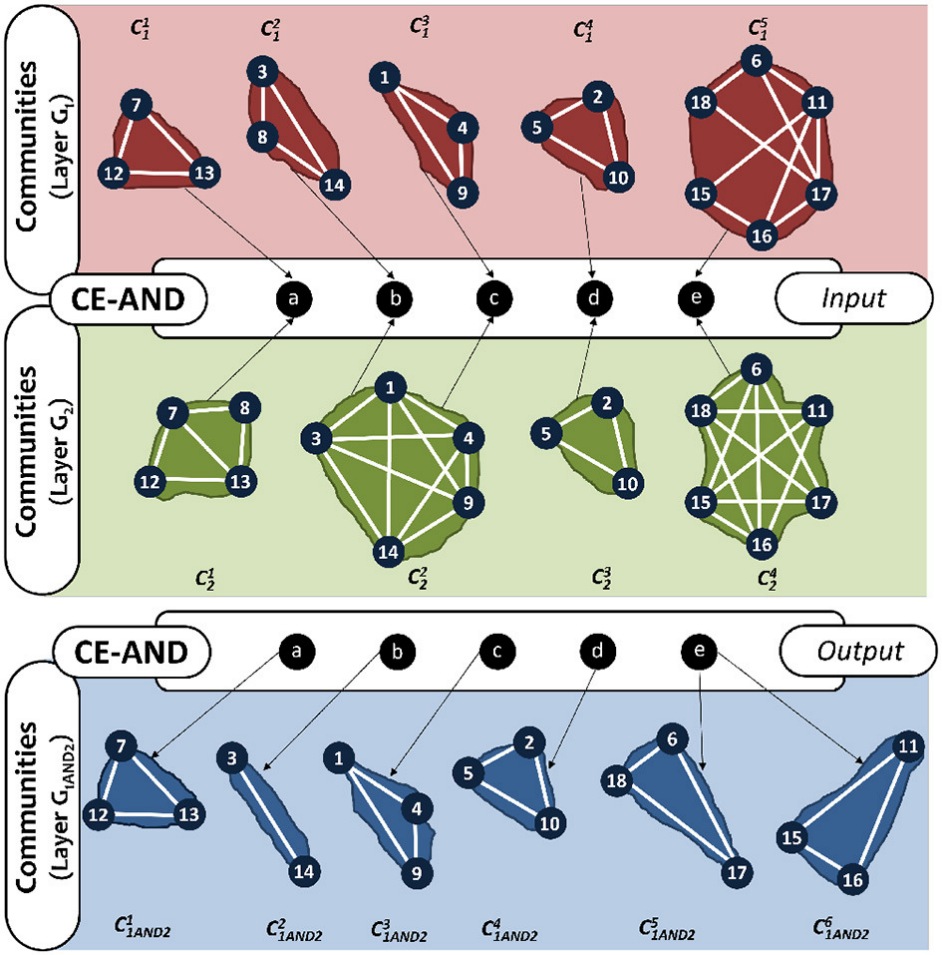
AND-composition communities of the multiplex in Figure 2, using CE-AND method.

We address these limitations by developing a community detection method, CE-AND (see Algorithm 5), that is based on the intersection of *edges* rather than *vertices* as follows. Here we consider pairs of communities, one from the first layer and the other from the second layer, and obtain the new community by taking the common edges among the pair. For every pair of communities, $C_{i}^{m}\left(V_{i}^{m},E_{i}^{m}\right)$ from layer *G*_*i*_ and $C_{j}^{n}\left(V_{j}^{n},E_{j}^{n}\right)$ from layer *G*_*j*_, the edge-based community intersection, $E_{i}^{m}\cap E_{j}^{n}$, will produce k disconnected edge-sets, $E_{iANDj}^{1}$, $E_{iANDj}^{2}$, ..., $E_{iANDj}^{k}$. These edge sets will form the AND-composed communities, $C_{iANDj}^{1},C_{iANDj}^{2},...,C_{iANDj}^{k}$. Figure 5 shows how the communities are obtained for the example network using CE-AND. Comparing this result to that in Figure 4, we see that most of the communities are obtained with the exception of the singleton node 8. The common bride edge (4, 5) is also missing.

**Algorithm 5:** Algorithm for CE-AND.

**Require:** Communities from layers *G*_*i*_ and *G*_*j*_: COMM(*G*_*i*_) = {$C_{i}^{1}\left(V_{i}^{1},E_{i}^{1}\right)$, $C_{i}^{2}\left(V_{i}^{2},E_{i}^{2}\right)$,..., $C_{i}^{x}\left(V_{i}^{x},E_{i}^{x}\right)$}, COMM(*G*_*j*_) = {$C_{j}^{1}\left(V_{j}^{1},E_{j}^{1}\right)$, $C_{j}^{2}\left(V_{j}^{2},E_{j}^{2}\right)$,..., $C_{j}^{y}\left(V_{j}^{y},E_{j}^{y}\right)$}
**Ensure:** return $L_{i,j}^{CE-AND}$ - a list of communities
1: $L_{i,j}^{CE-AND}$ = ∅ {Initialize the set of communities to an empty set.}
2: **for** each community pair say, $C_{i}^{p}$ and $C_{j}^{q}$ **do**
3: {$C_{i,j}^{p,q}$} = ($E_{i}^{p}$ ∩ $E_{j}^{q}$) {Create list of k new communities by taking the common **edges** of every pair of communities.}
4: $L_{i,j}^{CE-AND}=L_{i,j}^{CE-AND}\cup \{C_{i,j}^{p,q}$} {Add new communities to the set of communities.}
5: **end for**

#### 3.1.4. Proof of correctness

Algorithms 3, 5 produce a set of disjoint clusters. Algorithm 1 produces a set of communities in the AND-composed network. We consider these communities as the ground truth. We *label an edge* as *internal* (if both end points are in the same community) and *external or bridge* otherwise.

We assume that the communities in the individual layers and the composed network have high clustering coefficients. That is, we do not consider accidental communities such as an edge or a line graph, that are formed due to an algorithmic artifact rather than the structure of the network. If such trivial communities are formed, we consider each vertex in them as a singleton community. The clusters formed by the intersection algorithms do not have this restriction, since they are not obtained using community detection algorithms.

We now present a proof of how well the clusters obtain by Algorithm 5, correspond to the ground truth communities. Let the set of communities obtained from the composed network be Γ. Let the set of clusters obtained using the CE-AND algorithm be Ψ.

Lemma 3.1. For any given cluster *X*∈Ψ, there will exist a set of communities $\left\{C_{1}^{X},\ldots C_{m}^{X}\right\}$, where $C_{i}^{X}\in \Gamma$, 1 ≤ *i* ≤ *m*, that form a partition of the vertices in *X*, if and only if, the set of edges common to all layers have the same label in all the layers.

*Proof.* We first prove the condition that if the common edges have the same label in all the layers, then the set of the union of vertices in $\left\{C_{1}^{X},\ldots C_{m}^{X}\right\}$ will form a partition of the vertices in *X*∈Ψ.

Let the set of vertices belonging to the cluster *X* be *U*_*X*_. Let the set of vertices belonging to community $C_{i}^{X}$ be $V_{i}^{X}$, and $\cup_{i=1}^{i=m}\left(V_{i}^{X}\right)=V_{X}$, i.e., the union of these vertices in *V*_*X*_. Since the communities are disjoint to prove that *V*_*X*_ is a partition of *U*_*X*_, we have to prove that *V*_*X*_ = *U*_*X*_.

It is easy to show that there exists a set of communities such that *U*_*X*_ ⊆ *V*_*X*_. We simply select the communities such that all vertices in *U*_*X*_ are included.

We prove *V*_*X*_⊆*U*_*X*_ by contradiction. Let *v* be a vertex that is in set *V*_*X*_ but not in *U*_*X*_. Since CE-AND retains all the common internal edges, and *v* is not in *U*_*X*_, therefore *v* will be connected to its neighbors in *V*_*X*_ by one or more external (or bridge) edges. Since we assume that all common edges have the same labels, therefore in none of the layers *v* is tightly connected to any subset of *V*_*X*_. Moreover, the communities in the composed network have high clustering co-efficient (or are singletons). Since *v* is not tightly connected to vertices in *V*_*X*_ it cannot be part of the community. Thus our assumption was wrong, and *V*_*X*_ ⊆ *U*_*X*_. Taken together, *V*_*X*_ ⊆ *U*_*X*_ and *U*_*X*_ ⊆ *V*_*X*_; thus *U*_*X*_ = *V*_*X*_, and *V*_*X*_ is a partition of *U*_*X*_.

For the only if part we show that if the common edges do not have the same labels in all the layers, then there may not exist a set of communities that form a partition of the vertices in a given cluster.

**Figure 6:**
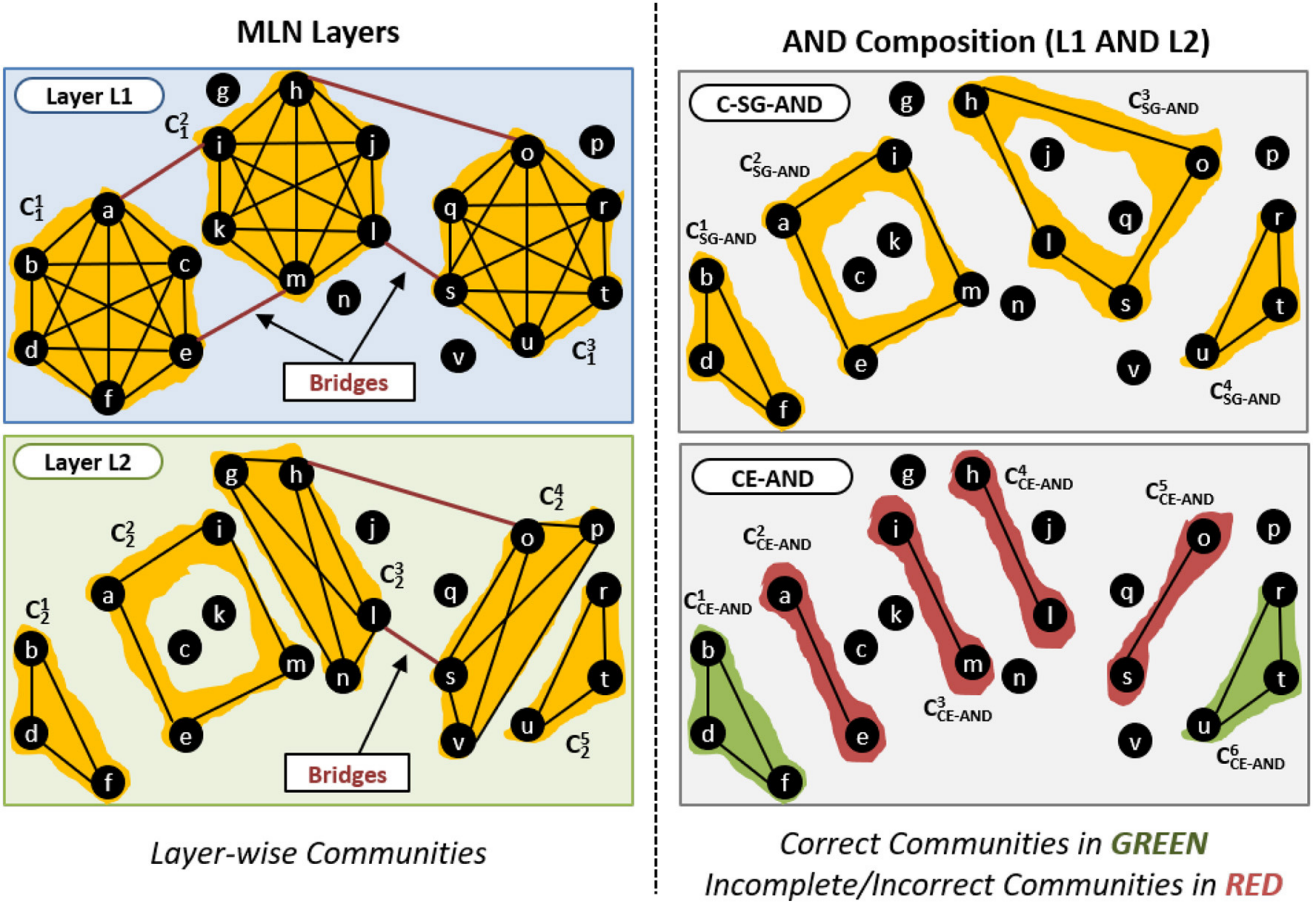
Effect of bridge edges on AND composition.

We provide such an example in Figure 6. The left-hand panels of Figure 6 shows two layers. The top right panel shows the communities obtained by the standard single network approach (C-SG-AND). The bottom right panel shows the communities obtained by CE-AND.

The community $C_{SG-AND}^{3}$ produced by C-SG-AND contains the edges (h, o) and (l, s) that act as bridges in Layer L1 and L2. CE-AND is not able to detect this community and instead produces two communities, $C_{CE-AND}^{4}$ and $C_{CE-AND}^{5}$, which should be merged into one by taking the bridge edges into account.

Also consider the community $C_{SG-AND}^{2}$ which consists of the edges (a, i) and (e, m) that are bridges in Layer L1, but are part of the community $C_{2}^{2}$ in Layer L2. As only those edges that are within community *in all layers* are considered, CE-AND produces two communities, $C_{CE-AND}^{2}$ and $C_{CE-AND}^{3}$.    □

### 3.2. Community detection in composed networks formed using the OR operation

We now consider how to obtain communities in composed networks formed using the OR operation (termed as *OR composed networks*). The number of edges in the OR-composed network is the union of the edges in each layer. For any two layers *G*_*i*_ and *G*_*j*_, the total number of edges is |*E*_*i*_∪*E*_*j*_|.

The computational complexity of community detection algorithms are at least proportional to the size of the graph. The denser the graph, the more time will be required to find the communities. Thus for the OR-composed case, our goal is not only to lower the time by reducing the need to recompute different compositions of layers, but also to reduce the size of the graph to be analyzed.

**Figure 7:**
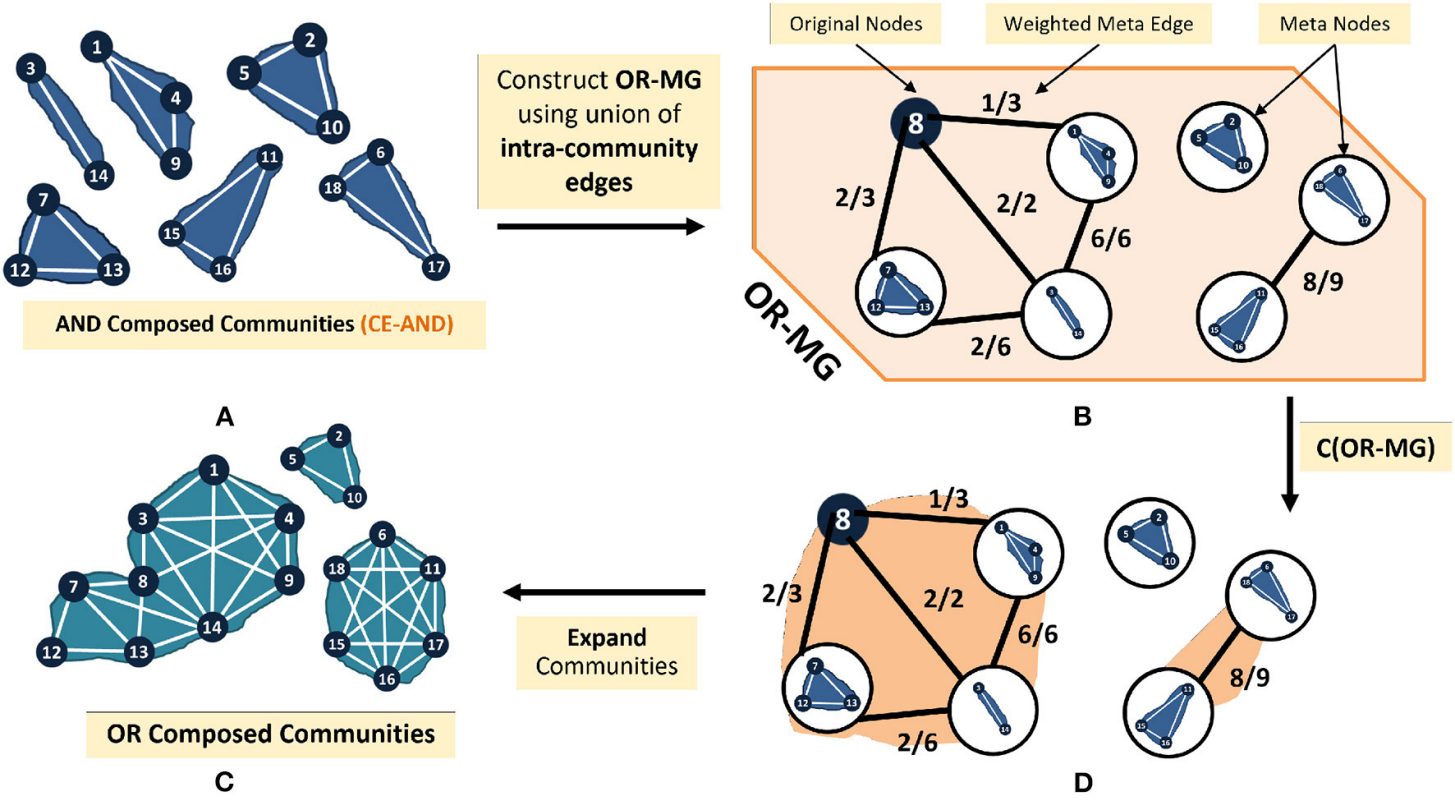
Illustrative flow **(A–D)** of CE-OR algorithm on the example graph.

To obtain communities of OR Composed Layers, we propose the CE-OR algorithm (Algorithm 6; Figure 7). This method reduces the size of the graph to be analyzed by processing the common communities as a single node. The steps of the CE-OR algorithm are as follows;

**Algorithm 6:** Algorithm for CE-OR.

**Require:** Communities from layers *G*_*i*_(*V, E*_*i*_) and *G*_*j*_(*V, E*_*j*_): COMM(*G*_*i*_) = {$C_{i}^{1}\left(V_{i}^{1},E_{i}^{1}\right)$, $C_{i}^{2}\left(V_{i}^{2},E_{i}^{2}\right)$,..., $C_{i}^{x}\left(V_{i}^{x},E_{i}^{x}\right)$}, COMM(*G*_*j*_) = {$C_{j}^{1}\left(V_{j}^{1},E_{j}^{1}\right)$, $C_{j}^{2}\left(V_{j}^{2},E_{j}^{2}\right)$,..., $C_{j}^{y}\left(V_{j}^{y},E_{j}^{y}\right)$}
**Ensure:** return $L_{i,j}^{CE-OR}$ - a list of communities { Find common communities using CE-AND}
1: Apply CE-AND on *COMM*(*G*_*i*_) and *COMM*(*G*_*j*_) to get $L_{i,j}^{CE-AND}$ **Construct** *OR-MG*(*V*_*OR*−*MG*_, *E*_*OR*−*MG*_) {Assign nodes of each common community as a meta node}
2: **for** each community *C*_*k*_(*U*_*k*_, *E*_*k*_) ∈ $L_{i,j}^{CE-AND}$ **do**
3: *V*_*OR*−*MG*_ = *V*_*OR*−*MG*_ ∪ *U*_*k*_
4: **end for** { Assign the vertices not in any common community as a meta node}
5: **for** each vertex *u*∉*C*_*k*_ $,\forall C_{k}\in L_{i,j}^{CE-AND}$ **do**
6: *U*_*k*_ = ϕ {Create null set}
7: *U*_*k*_ = *U*_*k*_∪*u* {Add *u* to the set}
8: *V*_*OR*−*MG*_ = *V*_*OR*−*MG*_ ∪ *U*_*k*_
9: **end for** {Add Edges in the metagraph. Two metanodes, (*U, V*) are connected if there is an intra-community edge from one constituent node of *U* to a constituent node of *V* in any one of the layers.}
10: **for all** all metanode pairs (*U, V*)∈*V*_*OR*−*MG*_ **do**
11: **if** ∃ *u, v, r*: (*u, v*) ∈ $E_{i}^{r}$ or (*u, v*) ∈ $E_{j}^{r}$, *u* ∈ *U* and *v* ∈ *V* **then**
12: *E*_*OR*−*MG*_ = *E*_*OR*−*MG*_∪(*U, V*)
13: **end if**
14: **end for**
15: Insert weights on the edges of OR-MG
16: L = COMM(OR-MG)
17: Expand the *community representative nodes* in each community from L to get $L_{i,j}^{CE-OR}$

#### 3.2.1. Overview of CE-OR

Find the common communities in all the network layers (Line 1) by using CE-AND. Then construct a metagraph (OR-MG), as follows. Each metanode represents *a set* of vertices. Combine the vertices of each common community into a metanode (Line 2–4). Vertices that are not assigned into communities are each separately assigned to a metanode (Line 5–9). Connect two metanodes, *U* and *V* via a metaedge, if there exists an internal edge, in *at least one* of the layers between an element (node) of *U* and an element (node) of *V* (Line 10-14). Apply appropriate weights to these edges (Line 15). Apply community detection on the metagraph (Line 16). The communities in the OR-composed network are obtained by expanding the metanodes in the communities obtained by the CE-OR algorithm.

#### 3.2.2. Assigning weights to metaedges

The metanodes represent vertex sets of varying sizes, and the number of edges between them represent the degree of similarity. Although the original graph is unweighted, the edges in the metagraph are weighted to quantify the extent of this similarity. A critical component of the CE-OR algorithm is based on correctly assigning these weights. We connect two meta nodes only if at least one pair of vertices from each meta node are connected by an internal edge, *in at least one of the layers*.

For any meta edge (*A, B*), let *V*_*A*_ and *V*_*B*_ be the set of nodes in the meta communities *A* and *B*, respectively. Further, let the set of all edges (internal with respect to at least one layer) between *V*_*A*_ and *V*_*B*_ be *E*_*A, B*_. We use the following two strategies to compute the weight of the metaedge;

- *Aggregation* is the number of edges between the two communities; *w*_*a*_(*A, B*) = |*E*_*A, B*_|
- *Fractional* is the fraction of connected nodes between the two communities; $w_{f}\left(A,B\right)=\frac{\left|E_{A,B}\right|}{\left|V_{A}|*|V_{B}\right|}$.

Figure 7 illustrates how the CE-OR algorithm is applied to the OR-composed layers of the IMDb graph. First the CE-AND communities obtained in Figure 5 and the vertex *I*_8_ are used to form the metanodes (Figure 7A). These nodes are connected based on the internal edges. The edges are weighted using *w*_*f*_ (Figure 7B). A community detection algorithm on the metagraph produces the communities of the OR-composed layers (Figure 7C). Comparing with the communities obtained by the C-SG-OR method in Figure 4, to those obtained by expanding the communities in the metanodes (Figure 7D), we see that all the communities have been obtained. However, the bridge edges between the communities are missing.

#### 3.2.3. Proof of correctness

We prove the correctness of our proposed CE-OR algorithm, by comparing the communities obtained by CE-OR to those obtained by executing community detection on the composed network. We define a metanode cluster, *Y*, as all the metanodes in a connected component of the metagraph. Let the communities obtained through the C-SG-OR algorithm be Λ.

Lemma 3.2. For a given metanode cluster *Y*, there will exist a set of communities $\left\{C_{1}^{Y},\ldots C_{m}^{Y}\right\}$, where $C_{i}^{Y}\in \Lambda$, 1 ≤ *i* ≤ *m*, such that $\left\{C_{1}^{Y},\ldots C_{m}^{Y}\right\}$ forms a partition of the vertices in *Y*, if and only if, all the internal edges of the communities in Λ were internal edges in at least one of the layers.

*Proof*. Let *U*_*K*_ be the set of vertices belonging to the metanode cluster *Y*, and let *V*_*K*_ be the union of the vertices in the communities $\left\{C_{1}^{Y},\ldots C_{m}^{Y}\right\}$. For the if direction it is sufficient to prove that *V*_*K*_ = *U*_*K*_.

*U*_*K*_ ⊆ *V*_*K*_, can be easily obtained by selecting the communities to form *V*_*K*_ such that all vertices of *U*_*K*_ are included. To prove *V*_*K*_ ⊆ *U*_*K*_ by contradiction, we assume that there exists a vertex *u*∈*V*_*K*_, that is not in *U*_*K*_. This means that *u* is connected to at least one vertex in *V*_*K*_ by bridge edges (or not connected at all). Thus at least one of the communities has an internal edge that was bridge edge in all the layers. This goes against our criteria that all internal edges for communities in the composed network, should be internal in *at least one* of the layers. Thus our assumption is wrong and *V*_*K*_ ⊆ *U*_*K*_. Since the communities are disjoint and *U*_*K*_ = *V*_*K*_, thus the statement is proven.

To prove the only if direction, we show that if the communities in the OR-composed layers have internal edges that were bridge edges in all the layers, then there may not exist a set of communities that form a partition for the vertices in a given metanode clusters.

**Figure 8:**
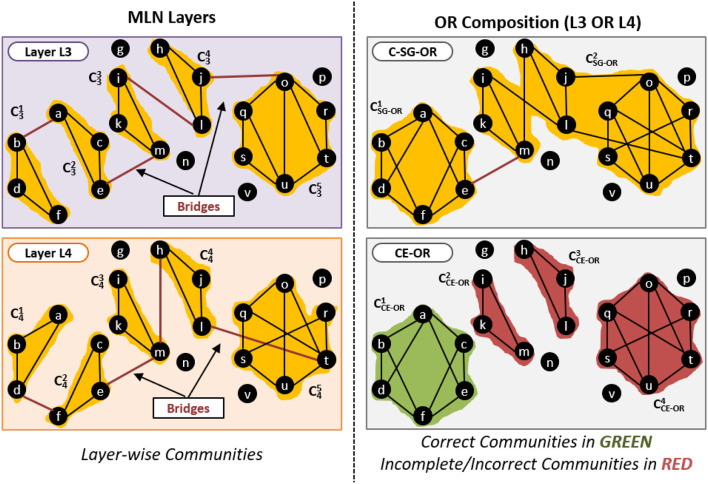
Effect of bridge edges on OR composition.

An example of this is given in Figure 8. The left-hand of panels show two layers of the network. The top right panel shows the communities obtained by the standard single network approach (C-SG-OR). The bottom right panel shows the communities obtained by our proposed CE-OR method.

Consider the community $C_{SG-OR}^{2}$ generated by C-SG-OR approach that has edges (i, l), (h, m), (j, o) and (l, t) which are not internal edges in any of the layers, and are present as bridge edges in only one of the layers. These edges will not be part of the metagraph and thus CE-OR does not know that they exist. CE-OR, thus, generates three communities $C_{CE-OR}^{2}$, $C_{CE-OR}^{3}$ and $C_{CE-OR}^{4}$, instead of merging them into one community, as per the C-SG-OR method.

However in the community $C_{SG-OR}^{1}$ generated by C-SG-OR, the edges (a, b) and (d, f) are bridge edges in one layer but are intra-community edges in another layer. Therefore these edges will be part of the metagraph. Thus CE-OR can use these edges and correctly generate the community $C_{CE-OR}^{1}$.

#### 3.2.4. Implications and limitations

The implication of Lemma 3.1 and Lemma 3.2 is that the CE-AND or CE-OR operations are successful if they create clusters that contain one or more communities in the composed networks in their entirety. Thus the communities can be divided into groups, such that each group can be mapped to exactly one cluster formed by the CE-AND or CE-OR operation. Therefore, the vertices in the composed network can be partitioned into subgraphs, with each subgraph relating to a cluster. Hence CE-AND and CE-OR operations are successful when each layer is formed of several loosely connected subgraphs, and bridges connecting the subgraphs do not change across the layers.

*The primary limitations* of our CE-AND and CE-OR algorithms is due to the non-inclusion of bridge edges. In the AND-composed network, we rationalize this non-inclusion by positing that communities formed solely of bridge edges cannot be dense, and hence are not strong communities. In the OR-composed network, we only exclude an edge if it is a bridge edge in *all* the layers.

### 3.3. CE-OR as a building block for pillar and semi-pillar communities

We demonstrate how CE-AND and CE-OR can be used as building blocks for the pillar and semi-pillar communities described in Section 2.2. Note that pillar and non-pillar communities are not dependent on how the networks/layers are combined, but on which communities are common across a given set of layers.

#### 3.3.1. Creating pillar communities

Since in pillar communities the same set of vertices form communities in every layer, therefore as per Lemma 3.1, the communities formed by CE-AND will be pillar communities. However, due to the strict restrictions of the CE-AND criteria, i.e. the edge has to be present in every layer, some vertices from pillar communities may be missed. To obtain these missing vertices, we then extend the communities found in CE-AND as per Algorithm 6 to form CE-OR communities. As per our experimental results in Section 4.2.2, we observe that CE-OR provides more accurate pillar communities.

#### 3.3.2. Creating semi-pillar communities

To find the semi-pillar communities, we take combination of all possible p layers from k layers, where 2 ≤ *p*<*k*. For each combination of layers we compute the communities using CE-AND. Note that since we use the decoupling method, therefore we need to compute the communities in each layer exactly once, and then combine the results. We identify the communities with the highest number of nodes, and extend them when possible using CE-OR. These communities across the layers form the semi-pillar communities.

## 4. Empirical results

We present the performance and accuracy of our CE-AND and CE-OR methods. First, we compare with the communities obtained from composed network created using AND/OR operations to merge the layers. Second, we compare with the ground truth pillar and semi-pillar communities given in Magnani and Rossi (2013). We use the popular community detection algorithm *Infomap* (Bohlin et al., 2014), both to find the communities in the single network approach (C-SG-AND, C-SG-OR) and the network decoupling approach(CE-AND, CE-OR). Our algorithms are implemented in C++ and were executed on a Linux machine with 8 GB RAM and installed with UBUNTU 16.10.

### 4.1. Comparison with communities on composed networks

*Data sets used*. We performed our experiments on multiplexes created from three real-world and one synthetic data set. To test on larger networks with more vertices, we created the synthetic data using the RMAT Chakrabarti et al. (2004) graph generator. The details of the data sets are as follows;

- *IMDb:* From the IMDb data set (IMDB-2018, 2018), the nodes in the multiplex represented the actors. In the first layer, (L1, co-acting) two nodes are connected if they co-acted in at least one movie. In the second layer, (L2, rating) two nodes are connected if the average ratings of their movies were similar. In the third layer, (L3, genre) two nodes are connected if they acted in movies of similar genres. For every actor, a vector was generated with the number of movies for each genre he/she has acted in. Two actors are connected if the Pearson's Coefficient between their corresponding genre vectors was at least 0.9^3^. Vertices:9,485; Edges in L1:45,581; Edges in L2: 13,945,912; Edges in L3:996,527.
- *DBLP:* From the DBLP data set of academic publications (dat, 2013), we selected all papers published from 2000 to 2018 in three conferences VLDB (L1), SIGMOD (L2) and ICDM (L3). Two authors (nodes) were connected if they had co-authored a paper for the conference corresponding to the layer. Vertices:17,204; Edges in L1:5,831; Edges in L2: 17,737; Edges in L3:12,986.
- *Accident:* From the data set of road accidents that occurred in the United Kingdom in 2014 (UKRoadData, 2014), two nodes (representing accidents) are connected in a layer if they occurred within 10 miles of each other and have similar Light (L1), Weather (L2) or Road Surface Conditions (L3). Vertices:5,000; Edges in L1:193,860; Edges in L2: 235,175; Edges in L3:216,397.
- *RMAT:* The RMAT generator creates networks based on the Kronecker product of a matrix. We set the number of vertices to 2^15^ and the edges to roughly eight times the number of vertices. We set the probabilities in each quadrant of the matrix as a=0.65, b=c=d=0.15 to create a scale-free graph.
  The first layer (L1) was the graph obtained by the generator. We applied cross perturbation to create layers (L2 and L3), as follows We selected two edges (a, b) and (c, d), and replaced them with new edges (a, c) and (b, d). Thus the number of edges and the degree distribution remain the same but the structure changes. In layer L2 we applied this perturbation to 1% of the edges and in layer L3 to 5% of the edges. Vertices:32,768; Edges in L1:230,445; Edges in L2: 230,445; Edges in L3:230,445.

*Ground Truth and Accuracy Metrics:* We use the communities obtained using C-SG-AND and C-SG-OR as the ground truth. We disregard singleton communities. We use two metrics to evaluate the accuracy of the communities - i) Normalized Mutual Information (NMI) that measures the quality with respect to the participating entity nodes only and ii) modified-NMI that also takes into account the topology of the communities. For both metrics higher is better, with maximum value of 1 and minimum of 0 [definitions in Labatut (2015)]. Each multiplex has 3 layers. Thus, a total of 4 compositions are possible (3 for 2-layers and 1 3-layers). We compare results for 8 (4 combinations X 2 Boolean operations) composed networks.

#### 4.1.1. Accuracy of the aggregation algorithms

**Figure 9:**
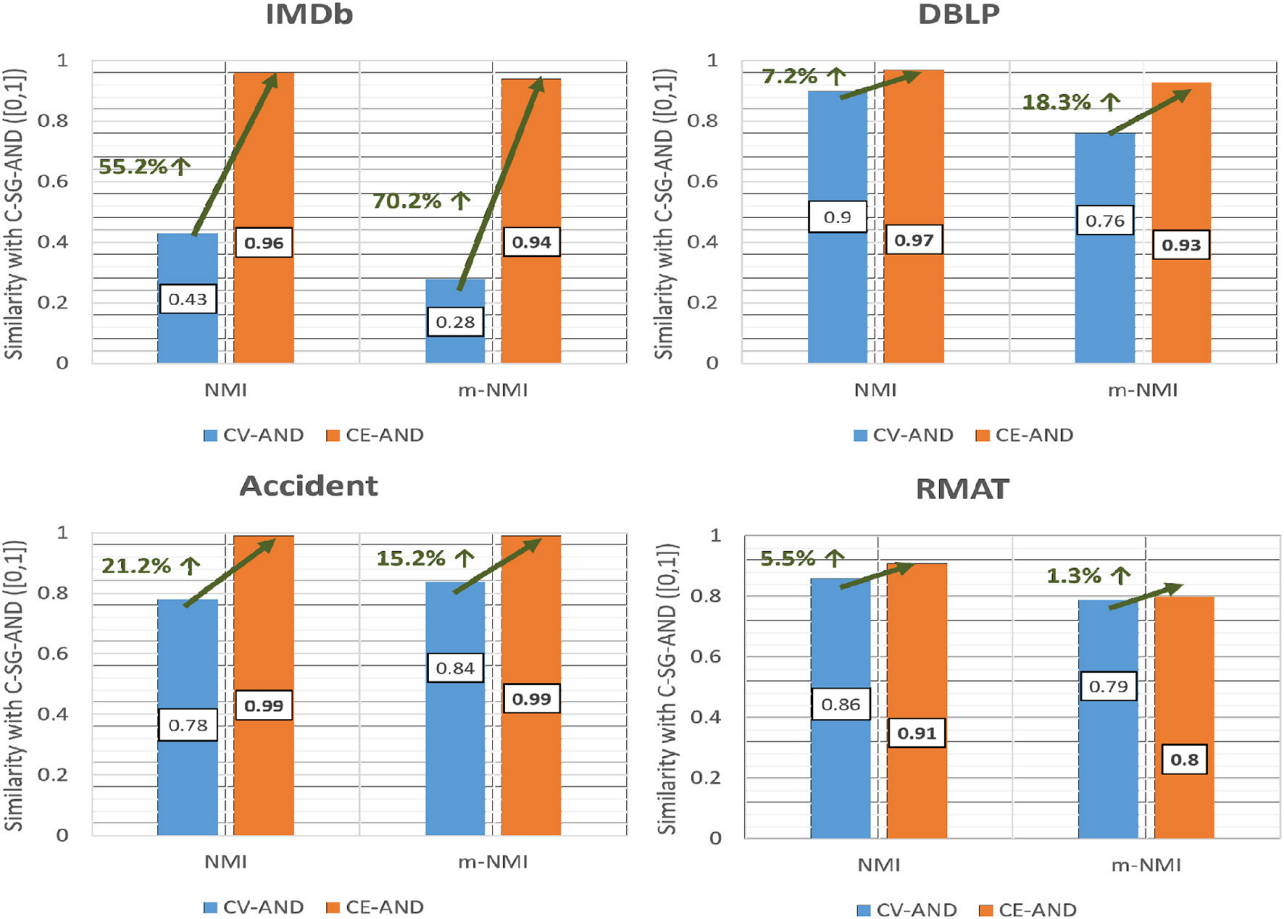
Comparison of accuracy of CE-AND and CV-AND based on NMI and m-NMI.

For the AND-composed networks we show in Figure 9, the average NMI and m-NMI of all the four multiplexes with respect to the ground truth for the CV-AND and CE-AND methods. The results show that the *accuracy obtained with CE-AND is higher than that from CV-AND*.

**Figure 10:**
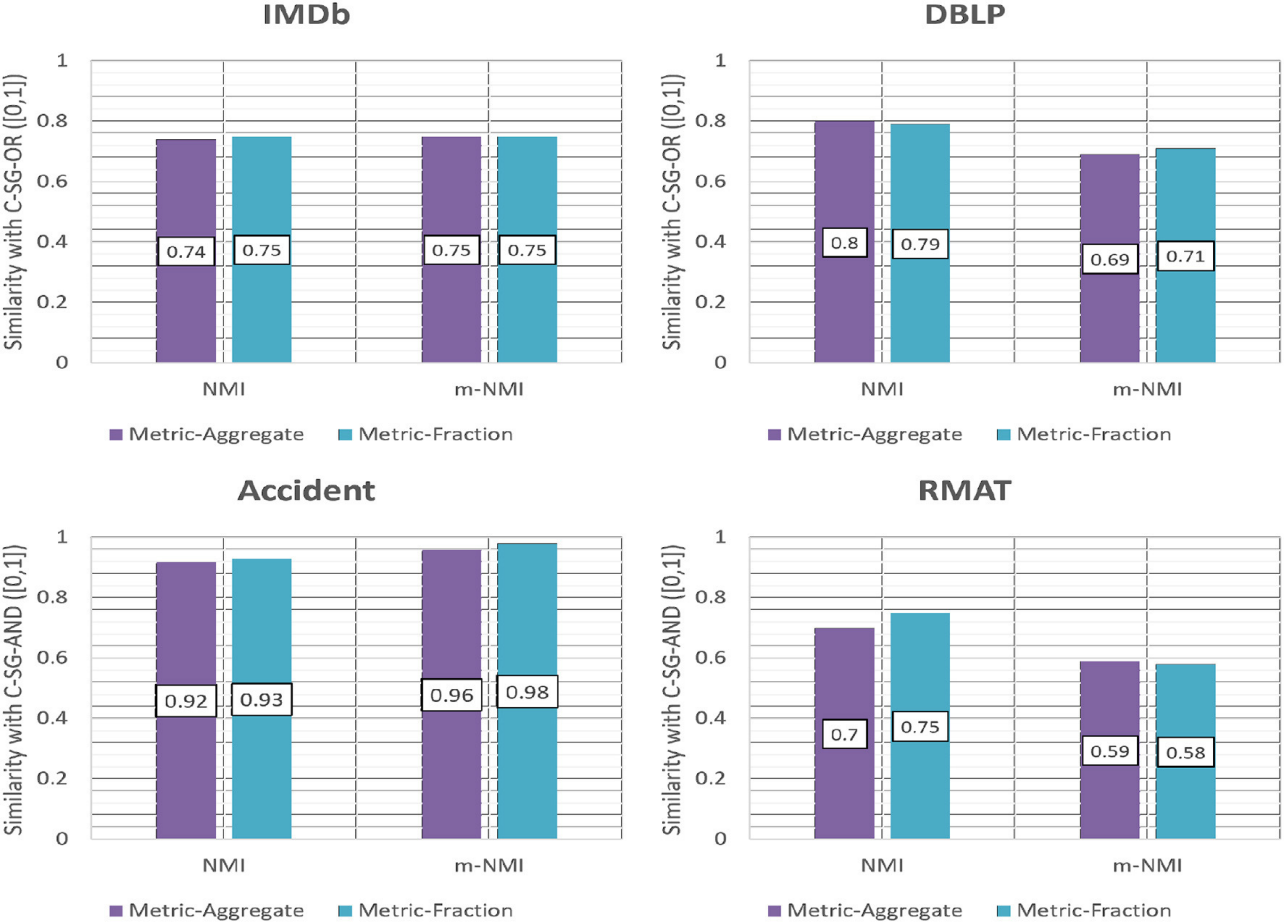
Accuracy of CE-OR with different weighting schemes based on NMI and m-NMI.

For the OR-composed networks we show in Figure 10, the average NMI and m-NMI of all the four multiplexes with respect to the ground truth for the two weighting metrics; Aggregation (*w*_*a*_) and Fractional (*w*_*f*_). The results show that the *accuracy obtained using both the metrics are similar*.

Table 1 shows the accuracy for the different multiplexes with respect to CE-AND for the AND composition and CE-OR with Fractional Weights. Nearly all the values are high, ≥70%. Some low values occur for the CE-OR method. An egregious example is IMDb (L1, L2) for which the accuracy results are less than 1%! In this case the metagraph had 193 nodes, and on running the community detection algorithm 56 communities were obtained. However, the ground truth communities obtained by C-SG-OR had only 2 communities. This happened because there existed many bridge edges in the layers that were not included in the metagraph. Moreover, because the communities represented in the metanodes were small in size, the edge weights were also lower and thus the communities could not combine.

**Table 1:** Accuracy Values using CE-AND and CE-OR on the different compositions of the data sets.

**Multiplex**	**L1, L2**		**L1, L3**		**L2, L3**		**L1, L2, L3**	
	**NMI**	**m-NMI**	**NMI**	**m-NMI**	**NMI**	**m-NMI**	**NMI**	**m-NMI**
**Accuracy Values using CE-AND**								
IMDB	0.97	0.93	0.98	0.97	0.88	0.86	0.99	0.99
DBLP	0.92	0.84	0.99	0.96	0.98	0.96	0.98	0.95
Accident	0.96	0.98	0.94	0.98	0.91	0.96	0.88	0.95
RMAT	0.92	0.82	0.90	0.79	0.90	0.78	0.90	0.77
**Accuracy Values using CE-OR using Fractional Weights**								
IMDB	< 0.01	< 0.01	0.97	0.99	1	1	1	1
DBLP	0.83	0.79	0.87	0.80	0.75	0.60	0.71	0.56
Accident	0.88	0.93	0.94	0.98	0.98	0.99	0.86	0.93
RMAT	0.74	0.64	0.76	0.59	0.75	0.55	0.73	0.54

#### 4.1.2. Performance of the aggregation algorithms

**Figure 11:**
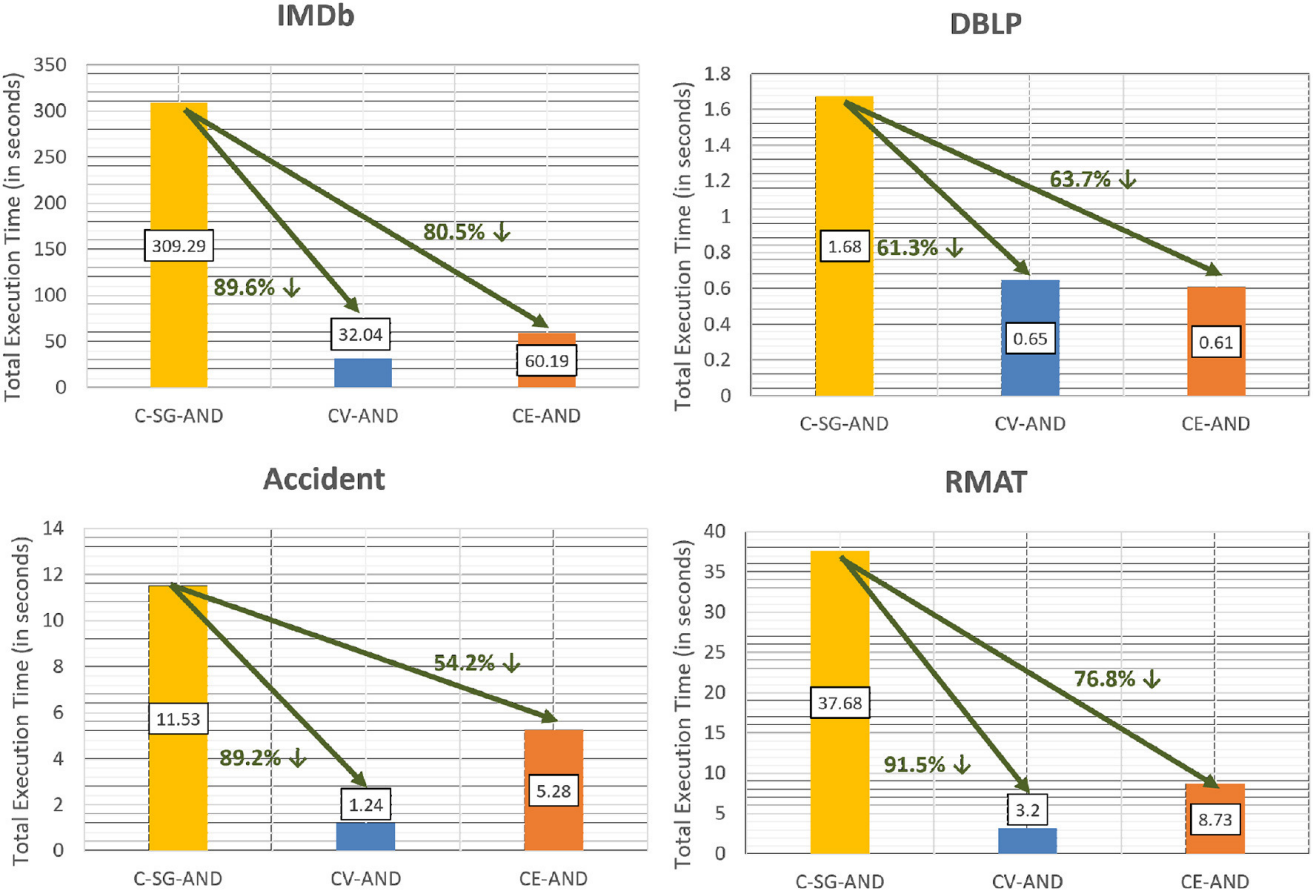
Efficiency of CV-AND and CE-AND as compared to C-SG-AND.

We now compare the time taken to obtain the communities using the aggregation methods (CV-AND, CE-AND and CE-OR) with respect to C-SG-AND and C-SG-OR. Figure 11 shows that the time to compute the communities over all the different composed layers is significantly lower for both CV-AND and CE-AND methods than C-SG-AND. When the layers are sparse, CE-AND will be faster than CV-AND, as can be seen for DBLP multiplex. However if the network layers are dense, then the edge-based intersection approach of CE-AND has a higher cost as compared to the CV-AND.

**Figure 12:**
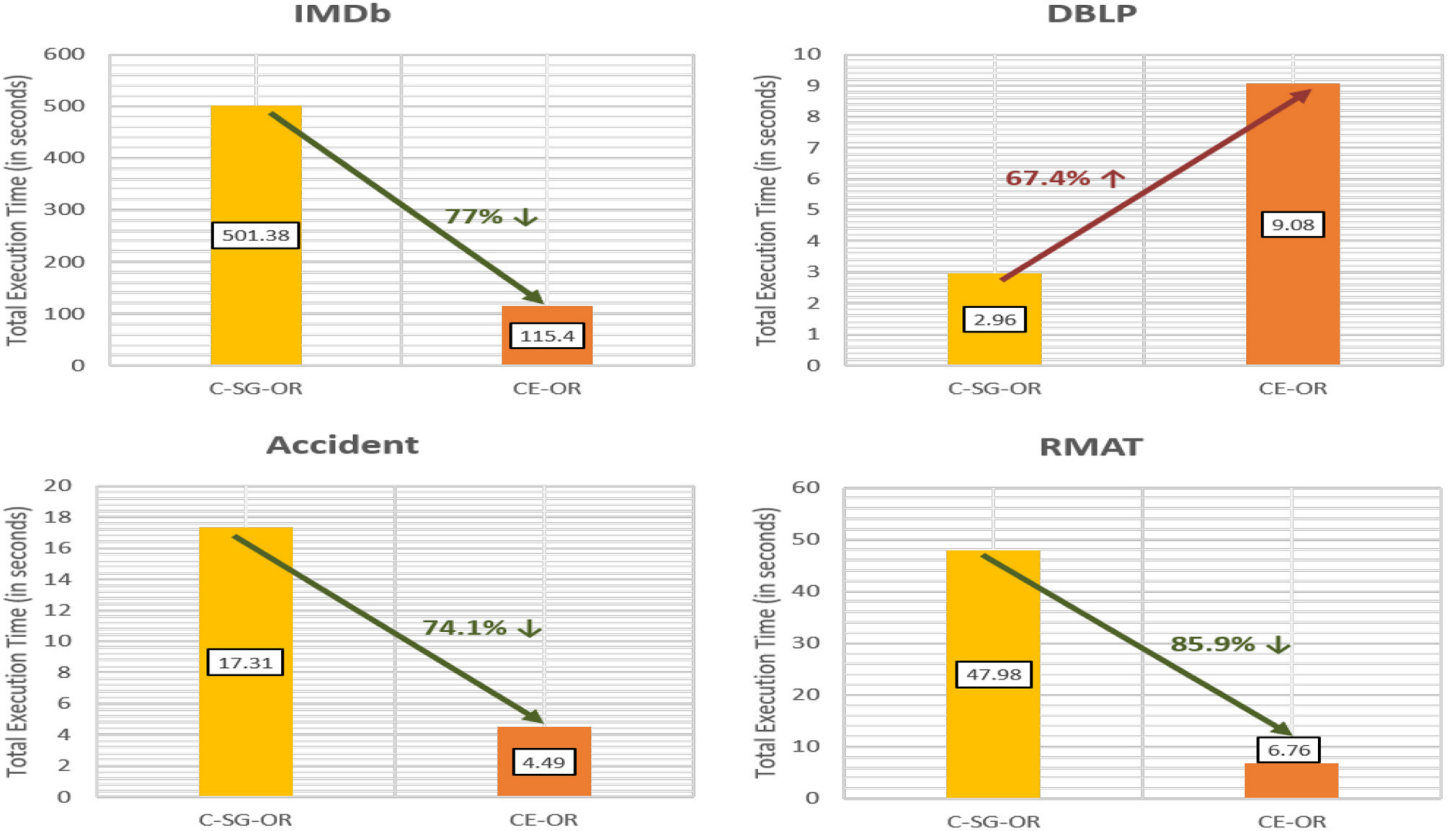
Efficiency of CE-OR as compared to C-SG-OR.

Figure 12 gives the time for executing CE-OR. For CE-OR, CE-AND is used as a subroutine. One scan of *community edges* is required to generate the meta graph (OR-MG) on which we apply Infomap. If the layers are sparse and the multiplex contains many bridge nodes, then cost of generating the meta graph and applying Infomap will become an overhead as compared to simply applying Infomap on OR graph (C-SG-OR approach). This can be seen from the DBLP multiplex where sparse layers (density of densest layer (SIGMOD) = 0.0001) make the CE-OR 67% less efficient as compared to C-SG-OR. However, *for multiplexes with fewer bridge edges (IMDb, Accident), CE-OR is significantly faster*.

### 4.2. Comparison with existing multilayer community detection algorithms

We compare the performance of our proposed decoupling based community detection algorithms against 16 community detection algorithms for multiplexes presented in (Magnani et al., 2021). We discuss how our multi-layer Boolean operation-based community definition can be used as a building block finding pillar and semi-pillar communities. We illustrate through experiments that our *CE-OR algorithm achieves a better accuracy* as compared to the baseline algorithms across a number of ground truth data sets.

#### 4.2.1. Ground truth data sets and metric

We use two real world data sets: AUCS and DKPOL and four synthetic data sets: PEP, PNP, SEP and SNP for calculating the accuracy of the proposed aggregation algorithms. Information about the real world data sets along with their ground truth community structure, as well as the code for generating the synthetic data sets and their communities are available in: https://bitbucket.org/uuinfolab/20csur/src/master/ (Magnani et al., 2021). The real world data set AUCS is 90 percent pillar partitioning, real world data set DKPOL and synthetic data sets PEP, PNP are 100 percent pillar partitioning. The synthetic data sets SEP and SNP have percentage of pillars column set to 0 since the data set is semi-pillar partitioning. More details are available in Magnani et al. (2021).

We use the *omega index* for comparing the results of our decoupling methods with respect to ground truth community structure for a data set. We select this metric to be consistent with the measures in Magnani et al. (2021). We evaluate the performance of our algorithm with respect to the existing algorithms in 4.2.2.

Omega index value is calculated by taking the mean of the number of agreements on two community sets *C*_1_ and *C*_2_ and normalizing by the expected number of agreements between the two community sets. When two nodes are present together in the same number of communities (j) in both community sets, it is called an agreement. The value of omega index ranges between 0 and 1. Here, 1 means two sets of communities are identical to each other. Formally, the omega index is computed as;

- $Omega\left(C_{1},C_{2}\right)=\frac{Observed\left(C_{1},C_{2}\right)-Expected\left(C_{1},C_{2}\right)}{1-Expected\left(C_{1},C_{2}\right)}$ where,
-
$Observed\left(C_{1},C_{2}\right)=\frac{1}{N}\overset{l}{\underset{j=0}{\sum }}\left(A_{j}\right)$

- $Expected\left(C_{1},C_{2}\right)=\frac{1}{N^{2}}\overset{l}{\underset{j=0}{\sum }}N_{j,1}N_{j,2}$ and,
- *l* = the maximum number of times a node pair appears together in both *C*_1_ and *C*_2_ at the same time,
- *N* = total number of possible node pairs,
- *A*_*j*_ = number of node pairs that are grouped together j times in both communities, and
- *N*_*j*, 1_, *N*_*j*, 2_ = number of node pairs that have been grouped together j times in *C*_1_, *C*_2_, respectively.

#### 4.2.2. Accuracy results

We have the ground truth communities available for real world and synthetic data sets. We apply our aggregation algorithms (CE-OR) on the data set AUCS which is 90 percent pillar, DKPOL, PEP and PNP which are 100 percent pillar, and SEP and SNP, which are semi-pillar.

We compared the accuracy of 17 community detection algorithms along with our proposed CE-OR aggregation algorithm over real world and synthetic data sets which have ground truth community structure available. The algorithms used are: flat_nw and flat_ec (Berlingerio et al., 2011), abacus (Berlingerio et al., 2013), cpm (Afsarmanesh and Magnani, 2016), glouvain (Mucha et al., 2010), infomap (De Domenico et al., 2015), scml (Dong et al., 2013), pmm (Tang et al., 2009) (Tang et al., 2012), lart (Kuncheva and Montana, 2015), emcd (Tagarelli et al., 2017), mlp (Boutemine and Bouguessa, 2017), and multiplex-leiden (Gurov et al., 2022). The results show that *on an average the CE-OR (Metric-Aggregate) algorithm has 89% accuracy and CE-OR (Metric-Fraction) algorithm has 82% accuracy*, which is significantly higher than the other methods.

**Figure 13:**
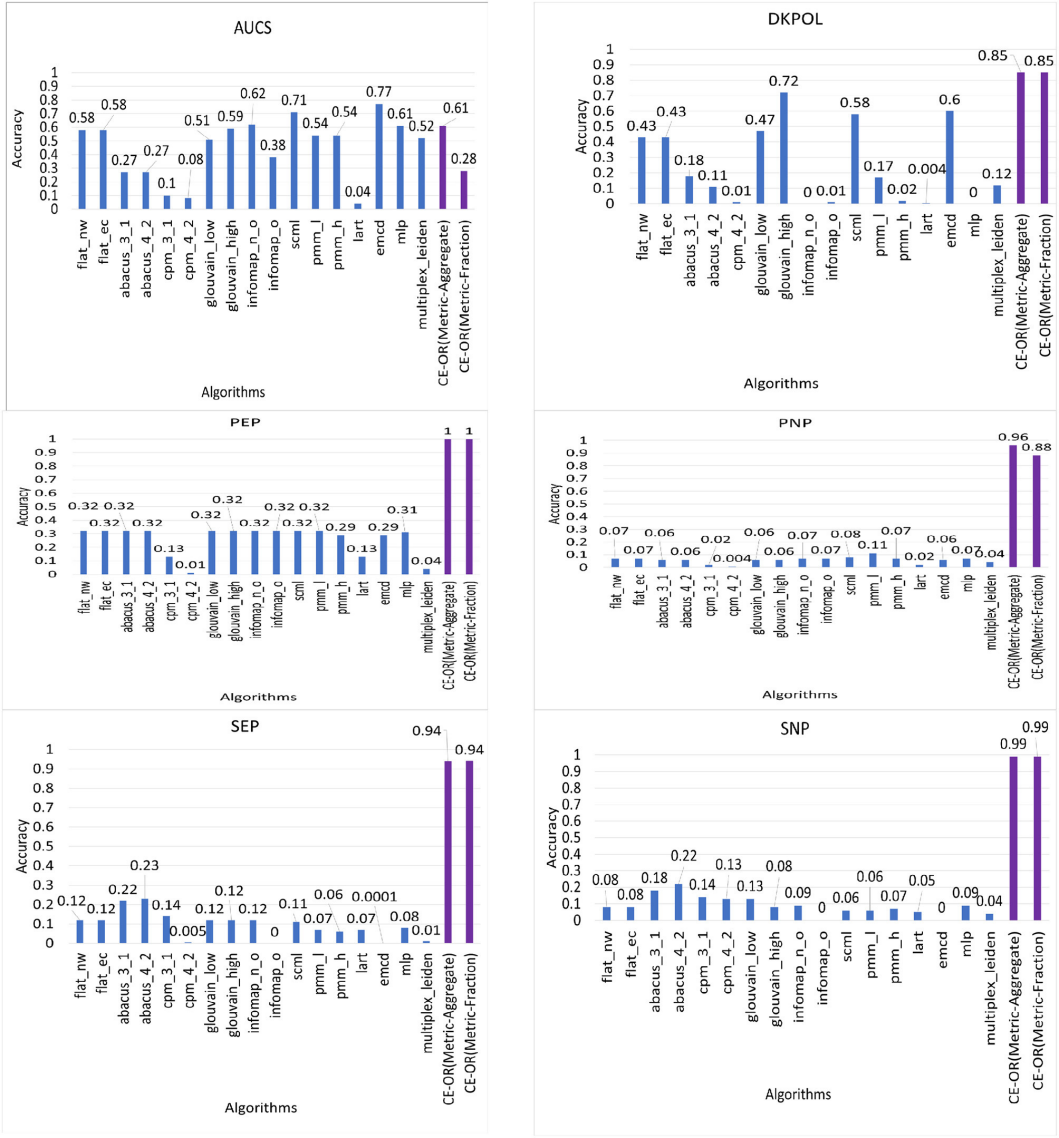
Accuracy (Omega index value) of different algorithms (existing algorithms along with CE-OR) for ground truth data sets.

In Figure 13, we present the *accuracy (omega index values)* values for the 16 existing community detection algorithms along with our proposed CE-OR aggregation algorithm for each data set. However, for AUCS data set, CE-OR does not perform better than many of the existing algorithms as AUCS does not have *complete* pillar partitioning. Our CE-OR algorithm improves the accuracy for data sets with 100% pillar partitioning community structure or known semi-pillar community structure. All other data sets with pillar partitioning the *accuracy of CE-OR ranges from 85% (DKPOL) to 100% (PEP)*.

## 5. Networks composed using extended boolean expressions

We now define the NOT composition, and demonstrate our proposed network decoupling methods can be used to efficiently and accurately analyze networks composed of a combination of Boolean expressions.

### 5.1. NOT composition

NOT of a layer will represent the *complement of the edge set* of that layer, i.e. the new layer will have all those edges that are *not* part of the original layer. Communities in a NOT layer will represent the groups of nodes that are *not strongly connected*. Examples of queries that can be answered using NOT are

- Groups of actors who have *not acted together* in a comedy (IMDb multiplex)
- Groups of authors who have *never co-authored* a paper in VLDB (DBLP multiplex)
- Groups of accidents that *did not have same Light condition* (Accident multiplex)

With respect to the single graph approach, these types of analysis can be handled by first generating the NOT layer and then applying community detection. AND, OR and NOT can be applied in different combinations, expanding the spectrum of analysis options. Although taking the complement of a layer is expensive and increases the number of edges in that layer, the cost depends on the graph density of the layer. Also, rewriting the expressions using the De Morgan's law can reduce the costs.

### 5.2. General boolean expression: accuracy, efficiency and drill down analysis

We demonstrate how general Boolean expressions can be computed using the decoupling approach. We use the DBLP multiplex with authors who publish papers at different conferences to address interesting analysis objectives. We consider all papers that were published from 2003 to 2007 in two high ranked conferences (VLDB and SIGMOD) and two medium ranked conferences (DASFAA and DaWaK). Based on whether two authors (nodes) have co-authored a paper in a particular conference, four layers were generated - all with the same 5116 vertices. VLDB (L1; Edges 3912; Communities 327), SIGMOD (L2 Edges 3303; Communities 254), DASFAA (L3 Edges 1519; Communities 229) and DaWaK (L4 Edges 679; Communities 154).

Few interesting analysis objectives that can be computed on the DBLP multiplex using Boolean expressions are as follows:

- Which collaborating groups who have published in *both the highly ranked conferences*, but have *never published in either of the medium ranked conferences*?
- Which co-author groups have only been able to publish in the low to medium rank conferences?
- Which author groups have published only in VLDB?

Based on the requirements of the analysis, it is important to figure out (a) the multiplex layers required and (b) the order in which the layers have to be composed using AND, OR, NOT. For the first analysis, “**Which are collaboration groups who have published in** ***both the highly ranked conferences*, but have** ***never published in either of the*** ***medium ranked conferences*?**”, we will compare the evaluation process for the traditional single graph approach and the proposed decoupling approach. *Single graph approach (SG)*: For the SG approach, the Boolean expression will correspond to ***SG:* COMM**[(VLDB *AND* SIGMOD) *AND NOT* (DASFAA *OR* DaWaK)]

This corresponds to first generating the required composed single graph and then applying the community detection algorithm to find the final set of communities. These communities acts as the ground truth. We used Louvain Blondel et al. (2008) to find the communities.

#### 5.2.1. Network decoupling approach

Using network decoupling, this expression will correspond to ***DEC1:* COMM**(VLDB) *CE-AND* **COMM**(SIGMOD) *CE-AND* **COMM** (*NOT* (DASFAA *OR* DaWaK))

That is, the layer-wise communities are composed to obtain the final set of communities. Alternatively, De Morgan's Laws can be applied to obtain another expression for the decoupling based boolean composition -

***DEC2:* COMM**(VLDB) *CE-AND* **COMM**(SIGMOD) *CE-AND* **COMM** (*NOT*(DASFAA)) *CE-AND* **COMM**(*NOT* (DaWaK))

We compare the efficiency of DEC1 and DEC2 with the single graph approach. We will evaluate the *NOT* (DASFAA *OR* DaWaK) by using the traditional OR of two layers and then take its complement. DEC2 uses the decoupling approach using operators CE-AND, and NOT as discussed in this paper. The layers of DBLP used above are *very sparse*, especially DASFAA and DaWaK. Hence, DEC2 will not be as efficient as DEC1 since it has to compute the complement of two layers (resulting in dense graphs) and then apply the decoupling approach. DEC1, on the other hand, has only one complement to compute.

#### 5.2.2. Accuracy results

For accuracy, the NMI and m-NMI values for the communities obtained by DEC1 and DEC2 have been compared against the communities obtained by SG. Both method, DEC1 and DEC2, provide more than 95% accuracy.

#### 5.2.3. Performance results

Both DEC1 and DEC2 resulted in the *same set of communities*. In DEC1, the number of CE-AND compositions are 2 whereas in DEC2 there are 3. Moreover, as the layers of the DBLP multiplex are sparse, their complement is dense. Thus, in DEC2 the Louvain is applied to two dense NOT layers. Thus, DEC2 will have a higher cost as compared to DEC1. Our results show that DEC1 is 2 times faster than DEC2. Therefore, it is very important to understand when to rewrite the expression (using De Morgans, Distribution, etc.) especially when the NOT operator is used on a composition of layers. *Finally, it is interesting to note that even with 2 dense graphs, DEC2 comes out better than the single graph approach. This further validates our decoupling approach even in the presence of NOT operator*.

#### 5.2.4. Drill-down analysis

**Figure 14:**
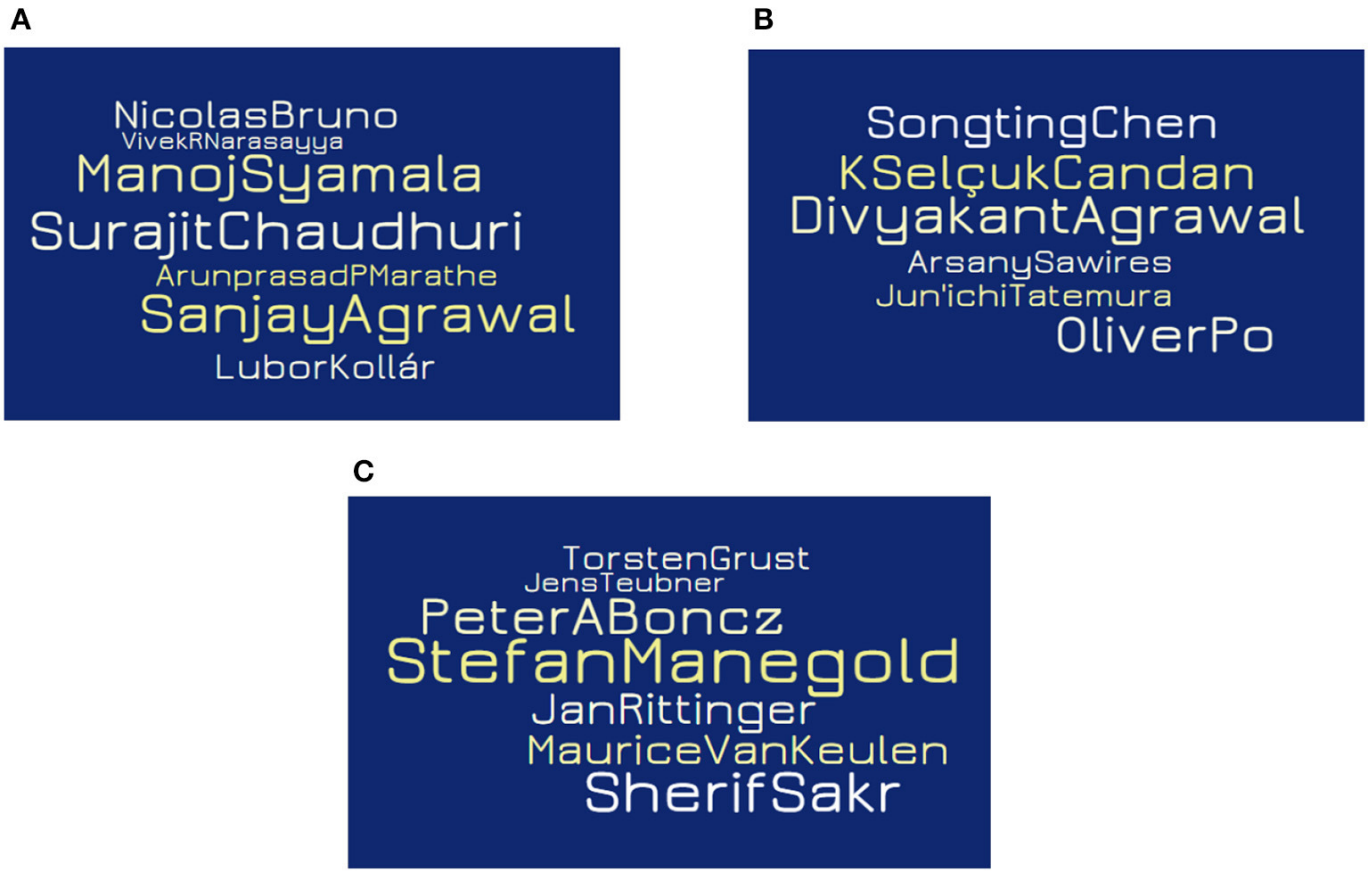
Drill-down analysis: prominent author groups. **(A–C)** represent author groups where most of them are published in VLDB and SIGMOD but never in DASFAA or DaWaK (time: 2003 to 2007).

One hundred and two communities are obtained from DEC1 and DEC2 that satisfy the requirement. Figure 14 shows few well-known groups most of whose members had collaborated on a paper that was published in both VLDB and SIGMOD, but never in DASFAA or DaWaK in the period from 2003 to 2007.

- Figure 14A community has researchers like **Surajit Chaudhari** who won the **VLDB 10-Year Best Paper Award (2007)** with **Vivek Narasayya** and **VLDB Best Paper Award (2008)** with **Nicolas Bruno**, apart from winning **ACM SIGMOD Contributions Award (2004)**.
- Figure 14B has researchers like **Divyakant Agrawal** who has **24000+ citations** (Google scholar).
- **Peter A. Boncz and Stefan Manegold** from Figure 14 (c) published a **highly cited paper** (350+ citations for MonetDB/XQuery) in SIGMOD 2006, and also won the **VLDB 10-year award**.

## 6. Related work

Recently, many analytical tasks have used multilayer networks to handle varying interactions among the same or different sets of entities such as co-authorship network in different conferences (Boden et al., 2012), citation network across different topics (Ng et al., 2011), interaction network based on calls/bluetooth scans (Dong et al., 2012) and friendship network across different social media platforms Magnani and Rossi (2013). Review of current work on multilayer networks are given in Boccaletti et al. (2014), Kivel et al. (2014), Kim and Lee (2015). Related software include Muxviz (De Domenico et al., 2014; Domenico, 2014), MAMMULT (Battiston et al., 2014; Nicosia and Battiston, 2015) and Pymnet (Kivel, 2018).

Community detection on a simple graph involves identifying groups of vertices that are more connected to each other than to other vertices in the network. Most of the work in the literature considers single networks where the objective is to optimize parameters such as modularity (Clauset et al., 2004) or conductance (Leskovec et al., 2008). As the combinatorial optimization of community detection is NP-complete (Brandes et al., 2003), a large number of competitive approximation algorithms have been developed (see reviews in Fortunato and Lancichinetti, 2009; Xie et al., 2013.)

Recently, community detection algorithms have been extended to Homogeneous MLNs (see reviews Fortunato and Castellano, 2009; Kim and Lee, 2015.) Algorithms based on matrix factorization Dong et al. (2012), cluster expansion philosophy (Li et al., 2008), Bayesian probabilistic models (Xu et al., 2012), regression (Cai et al., 2005) and spectral optimization of the modularity function based on the supra-adjacency representation (Zhang et al., 2017) and a significance based score that quantifies the connectivity of an observed vertex-layer set through comparison with a fixed degree random graph model (Wilson et al., 2017) have been developed. However, all these approaches *analyze a MLN either by aggregating all (or a subset of) layers of a HoMLN using Boolean and other operators or by considering the entire MLN as a whole*, leading to issues with respect to loss of information and computational inefficiency.

Recent works include Jin et al. (2019) using a Bayesian probabilistic model based on multiplex semantics to find communities in multiplex networks, DeFord et al. (2019) using spectral clustering approach and Li et al. (2023) using motifs for identifying higher-order interaction in each layer, and then agglomerateing the layers. A new algorithm named semidefinite programming (SDP) was proposed in Tang et al. (2023) that uses the node attributes and network structure information to identify communities on node-attributed networks and the multiplex network. The authors in Lyu et al. (2022) proposed a technique named evolutionary multiplex optimization to identify communities in a multiplex network that solves the problem of community detection in each layer as a multitask optimization problem.

To identify the communities in the temporal multiplex graph, the authors in Rebhi et al. (2021) proposed a two-step method to detect communities in a temporal multiplex network where the first step uses a new hybrid community detection algorithm to identify partition and the second graph identifies the final stable communities. Ideas like semi-aggregation have also been used in Kis and Gaskó (2020) where each layer is altered based on the structure of the layers on other layers. A new stochastic block model-based community detection algorithm is proposed in Liu et al. (2020) that uses a two-stage procedure avoiding the concept of same node membership. The authors in paper (Huang et al., 2019) propose a model based on the concepts of generic localized community label constraints, the Stochastic Block Model, and the Belief Propagation algorithm. The bayesian model has been found to effectively identify community structure from a multiplex network in Amini et al. (2022). A supervised algorithm based on layer convex flattening and modularity optimization of the network, as shown in Gurov et al. (2022), has been developed for community detection in multiplex networks.

Bio-inspired optimization has successfully solved the problem of community detection in network that we observe from the work Osaba et al. (2020) where the authors present in detail the problem of community detection from view of bio-inspired computation. Works like Al-sharoa and Rahahleh (2023) show a deep robust auto-encoder nonnegative matrix factorization (DRANMF) approach consisting of a deep structured decoder and encoder components to detect the community structure in networks.

## 7. Discussion and future work

We presented algorithms for efficiently finding communities in Boolean composed layers of multiplex networks. The results show that for most cases our algorithms are significantly faster than the standard methods and produce results of similar quality. The only cases that our algorithm fails is when the layers have significantly more bridge edges. We further demonstrated that our network decoupling methods can be used as building blocks for different types of multilayer communities in literature, such as pillar and semi-pillar communities. In these cases too, network decoupling produces results with higher accuracy compared to other baseline methods. The only case our method produces lower accuracy is when a network designated to have pillar communities does not have the complete pillar information. Given these results we can posit that network decoupling is an effcient and effective method for finding communities in homogeneous multilayer networks.

In future, we will investigate how to include some percentage of bridge edges without increasing the computation time. We also plan to explore adaptive techniques that can select between the network decoupling and standard methods as suitable. Finally, we also aim to develop methods to include meaningful NON-BOOLEAN combinations for weighted networks.

## Data availability statement

The original contributions presented in the study are included in the article/Supplementary material, further inquiries can be directed to the corresponding author.

## Author contributions

AS and FI conducted the experiments and wrote the paper. SC, SB, and KM helped in designing the experiments. SC and SB helped in writing and editing. All authors contributed to the article and approved the submitted version.

## Footnotes

^1^We state that the communities should be similar rather than identical, because community detection is non-deterministic, and even slight changes in the algorithm or order in which the vertices are processed can slightly alter the results.

^2^The notion of tightness can be quantified by density, modularity (Blondel et al., 2008), average flow (Bohlin et al., 2014), and so on. Additionally, self-preservation of communities can be verified using any well-established community detection algorithms like Infomap and Louvain.

^3^The choice of the threshold is based on how actors are weighted against the genres.
