# Supplementary figures and images for: Efficient community detection in multilayer networks using boolean compositions
Source: Front Big Data. 2023 Aug 23;6:1144793. doi: 10.3389/fdata.2023.1144793 (PMC10481956; doi:10.3389/fdata.2023.1144793)

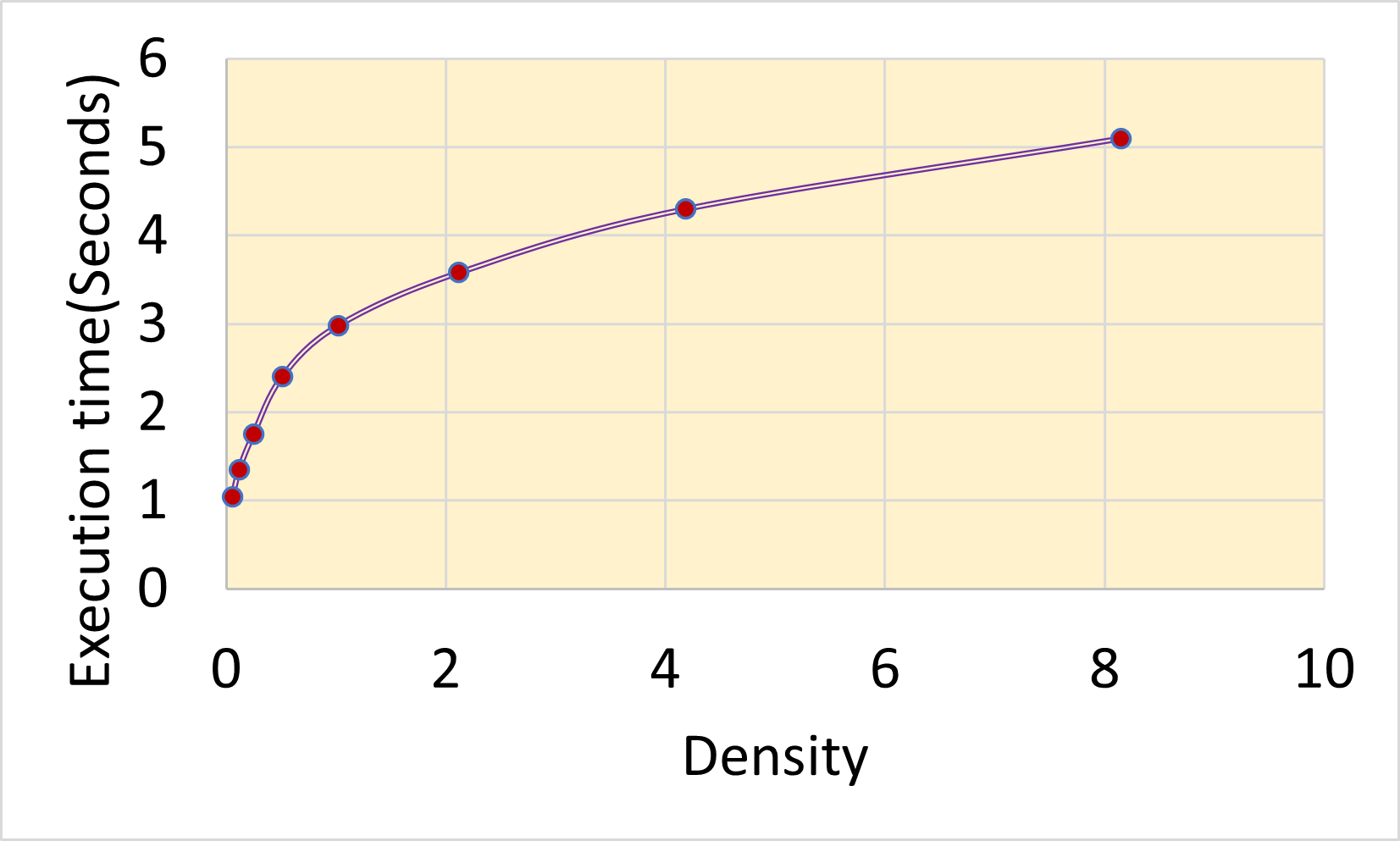

Supplement: Supplementary file 1 [file Image_1.PNG]

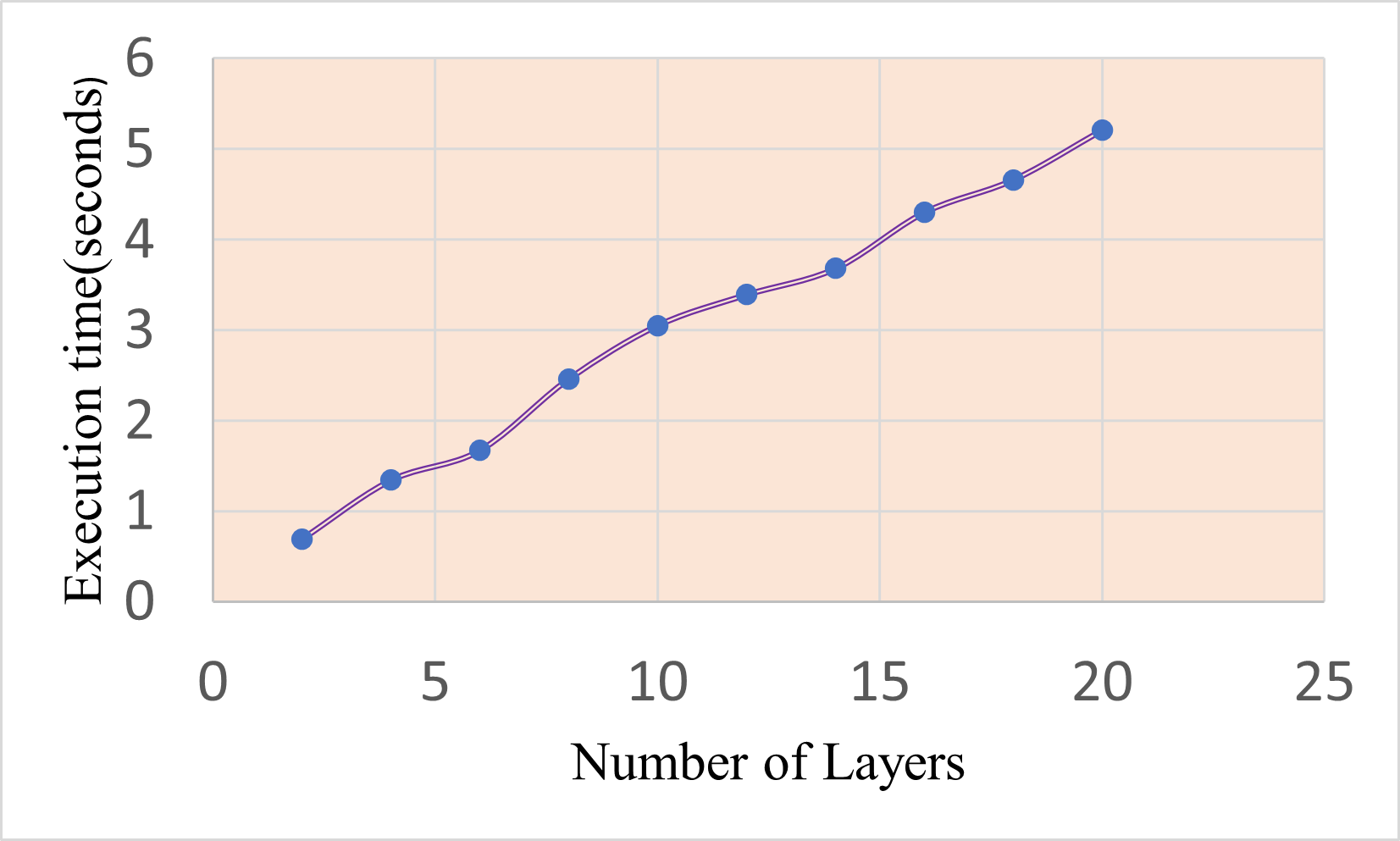

Supplement: Supplementary file 2 [file Image_2.PNG]

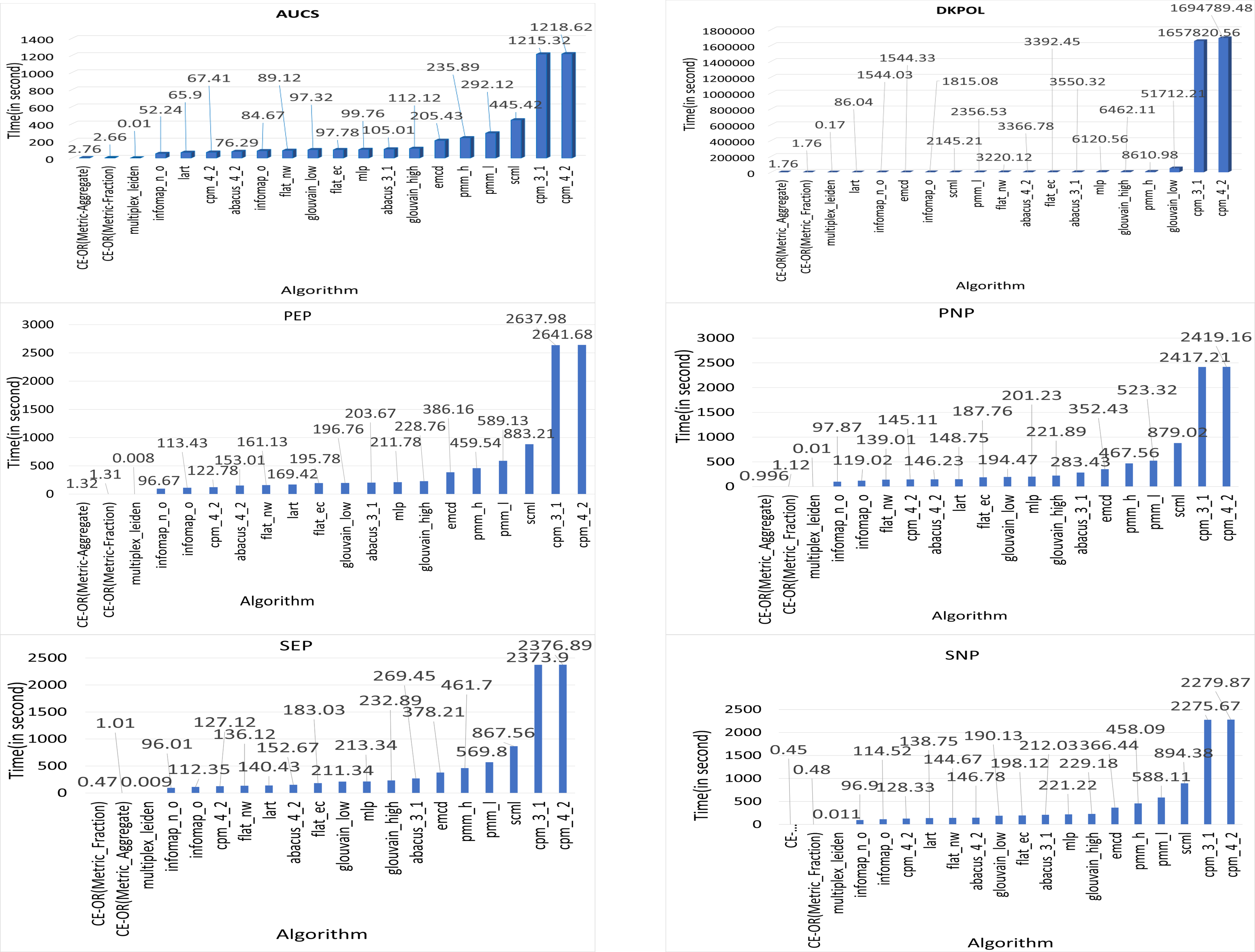

Supplement: Supplementary file 3 [file Image_3.PNG]
